# Sex Difference and Socioeconomic Inequity in Chinese People With Hypertension: National Cross-Sectional Survey Study

**DOI:** 10.2196/63144

**Published:** 2024-11-20

**Authors:** Xiaoyun Zhang, Siyu Wang, Qianqian Yang, Ruizhi Zheng, Long Wang, Hong Lin, Shuangyuan Wang, Mian Li, Tiange Wang, Zhiyun Zhao, Jieli Lu, Min Xu, Yuhong Chen, Jie Zheng, Meng Dai, Di Zhang, Weiqing Wang, Guang Ning, Yufang Bi, Yu Xu

**Affiliations:** 1 Department of Endocrine and Metabolic Diseases, Shanghai Institute of Endocrine and Metabolic Diseases, Ruijin Hospital, Shanghai Jiao Tong University School of Medicine, Shanghai, China; 2 National Clinical Research Center for Metabolic Diseases (Shanghai), Ruijin Hospital, Shanghai Jiao Tong University School of Medicine, Shanghai, China; 3 Key Laboratory for Endocrine and Metabolic Diseases of the National Health Commission, Ruijin Hospital, Shanghai Jiao Tong University School of Medicine, Shanghai, China; 4 National Research Center for Translational Medicine, Ruijin Hospital, Shanghai Jiao Tong University School of Medicine, Shanghai, China; 5 State Key Laboratory of Medical Genomics, Ruijin Hospital, Shanghai Jiao Tong University School of Medicine, Shanghai, China

**Keywords:** sex difference, socioeconomic inequity, blood pressure, hypertension, cross-sectional survey

## Abstract

**Background:**

Sex differences in blood pressure (BP) levels and hypertension are important and the role of socioeconomic status (SES) in sex differences in hypertension remains unclear.

**Objective:**

This study aimed to evaluate the impact of SES on sex differences of hypertension in a nationally representative survey study.

**Methods:**

A total of 98,658 participants aged ≥18 years who have lived in their current residence for ≥6 months were recruited from 162 study sites across mainland China. Sex was self-reported. Individual-level SES included the highest level of education and annual household income. Area-level SES included economic development status, urban/rural residency, and north/south location. Outcomes included levels of systolic and diastolic BP, and hypertension. Linear and Cox regression models were used to examine the associations between sex (women vs men) and BP characteristics stratified by individual or combined SES indicators.

**Results:**

Systolic and diastolic BP levels and the prevalence of hypertension were higher in men than in women. This sex difference was found across categories of SES with widened sex disparities in participants having more favorable SES. Significant multiplicative interaction effects of SES on the association of sex with BP characteristics were found. Women with improving SES were associated with lower BP and hypertension prevalence compared to men. For combined SES, a 9% (prevalence ratio 0.91, 95% CI 0.83-0.98) and a 30% lower probability (prevalence ratio 0.70, 95% CI 0.63-0.78) of having hypertension were found in women with an overall intermediate SES and high SES, respectively, compared to those with low SES, while no significant reduction was found in men.

**Conclusions:**

There are significant sex differences in BP characteristics and SES has a potent impact on the disparities. Sex-specific public health policies to alleviate socioeconomic inequalities, especially in women are important for the prevention of hypertension.

## Introduction

Hypertension is the major modifiable cause of premature death worldwide and in China. According to a recent survey, the standardized prevalence of hypertension reached 24.7% in 2018 and high systolic blood pressure (BP) was one of the leading risk factors contributing to deaths in mainland China [1,2]. Using the new hypertension definition of a systolic BP ≥130 mm Hg or a diastolic BP ≥80 mm Hg [3], the prevalence of hypertension in Chinese adults has nearly doubled (46.4%), whereas the awareness and control of hypertension remain substantially low [4].

Sex differences exist in hypertension, and it is widely known that men have higher BP levels and hypertension prevalence than women before the age of 60 years, after which the trend reversed between men and women [5,6]. Based on the Global Burden of Diseases, Injuries, and Risk Factors Study, the annual increase in global hypertension prevalence from 2000 to 2019 was only found in women but not in men, while hypertension-related mortality rate decreased more pronouncedly in women than in men [7]. In addition, previous studies have shown that unfavorable socioeconomic status (SES) is associated with increased hypertension risk [8-11]. However, most previous epidemiological studies reported the contribution of a single SES indicator, such as education or income without fully considering a comprehensive set of SES indicators, which might affect health in an aggregated way. Ignoring the synergistic effects of various social risk factors undoubtedly undermines efforts to identify socially disadvantaged people and implement targeted interventions to address social inequities [12]. Moreover, the role of SES in the sex differences of hypertension remains underinvestigated, and limited studies have explored the complex associations between SES, sex, and hypertension among general populations.

Therefore, using data from the China Noncommunicable Disease Surveillance study with a wide coverage of geographical areas and SES across mainland China, we aimed to evaluate the potential impacts of SES on sex differences in BP levels and prevalent hypertension in a general population.

## Methods

### Study Population

The China Noncommunicable Disease Surveillance study is a nationally representative cross-sectional survey of the general population aged ≥18 years in mainland China in 2010. Details of the study design and protocol have been described previously [13,14]. In brief, the study included all 162 study sites from the Chinese Center for Disease Control and Prevention’s (CDC’s) National Disease Surveillance Point System, covering major geographic areas of all 31 provinces, autonomous regions, and municipalities in mainland China. The first level of sampling was stratified by 7 geographic regions (northeast, north, east, south, southwest, northwest, and central areas) and 3 municipalities (Beijing, Tianjin, and Shanghai) in China. The second level of sampling was stratified by urban and rural locations. The third level of sampling was stratified by 4 socioeconomic strata in rural areas and 3 population-size strata in urban areas [15]. At each study site, a complex, multistage, probability sampling design was used to select participants who were representative of civilian, noninstitutionalized Chinese adults. Specifically, at the first stage of sampling, 4 subdistricts or townships at each study site were selected with probability proportional to size. In the second stage of sampling, 3 neighborhood communities or administrative villages within each subdistrict or township were selected with probability proportional to size. In the third stage of sampling, 50 households were randomly selected from each neighborhood community or administrative village. At the final stage of sampling, one person aged 18 years or older was selected using a Kish selection table from each household. Finally, a total of 98,658 individuals participated in the survey. The study protocol was approved by the Ethical Review Committee of China CDC and other participating institutes. Written informed consent was provided by each study participant. Participants or the public were not involved in the design, conduct, reporting, or dissemination plans of our study.

### Ethical Considerations

The study was conducted according to the guidelines of the Declaration of Helsinki, and approved by the ethical review committee of China CDC and other participating institutions (number 201010). All participants involved in the study provided written informed consent and participants were not compensated due to the observational design of the study. The data used in the analyses were entirely anonymous and deidentified for the protection of participants’ privacy and confidentiality.

### Data Collection

Data collection was conducted by trained staff at examination centers of local health stations or community clinics near participants’ residences following a standard protocol. A comprehensive face-to-face questionnaire was administered to collect demographic characteristics, SES, lifestyle factors, history of chronic diseases, current medications, etc. Current smoking was defined as having more than 100 cigarettes during a lifetime and currently smoking cigarettes. Current drinking was defined as drinking alcohol more than once per month during the past 12 months. The assessment of physical activity was based on the Global Physical Activity Questionnaire [16]. A food frequency questionnaire was used to record the dietary habits of typical food items in the past 12 months. A healthy diet score was calculated based on the recommendations of the American Heart Association [17] with modifications using soy protein to replace whole grain intake [14]. Height and weight were measured according to a standard protocol and BMI was calculated as weight in kilograms divided by height in meters squared. Obesity was defined as BMI ≥28 kg/m^2^ [18]. Waist circumference was obtained during a standing position measured as the length of the midway between the lower edge of the costal arch and the upper edge of the iliac crest. Participants were asked to avoid coffee, tea, alcohol, exercise, and cigarettes 30 minutes before BP measurement, during which 3 consecutive measurements were taken with a 1-minute interval between each measurement after a 5-minute sitting rest using an automated device (OMRON Model HEM-7071, Omron Co) with observers present. The average of 3 consecutive measurements was used for analysis.

### SES Indicators

We used both individual-level and area-level SES indicators in this study. Individual-level SES indicators included the highest level of education and annual household income. Area-level indicators included economic development status, urban/rural residency, and north/south location. Education level was based on self-report and categorized into 3 groups, including less than primary education (duration of education<6 y), primary and lower-secondary education (duration of education 6‐9 y), and upper-secondary education or above (duration of education>9 y). Annual household income was self-reported and was categorized into higher and lower income groups based on income levels above and below the median (20,000 Chinese Yuan [CNY;US $2954.4 according to the annual average CNY/US $ conversion rate in 2010] per year). Economic development status was determined based on gross domestic product (GDP) per capita in 2009 at the study site and categorized by tertiles into underdeveloped (GDP per capita <14,397 CNY as <US $2126.7), intermediately-developed (GDP per capita between 14,397 and 27,107 CNY as between US $2126.7 and US $4004.2), and developed (GDP per capita>27,107 CNY as >US $4004.2). Urban and rural residency was determined according to the National Bureau of Statistics of China [19]. North and south location of the study site was divided by the Qinling Mountains-Huaihe River Line across mainland China. We then assigned a score of 0 (for low SES) or 1 (for high SES) to each SES indicator and calculated a composite SES score for each participant by summing individual indicator scores up. Educational attainment and economic development were each dichotomized according to the median duration of education and median GDP per capita, respectively for the score. Therefore, the composite SES score ranged from 0 to 5, and participants were categorized into the overall low-SES group (0‐1 point), intermediate-SES group (2‐3 points), and high-SES group (4‐5 points) based on their composite SES scores.

### Hypertension Definition

Hypertension was defined by a self-report of using antihypertensive medications within the previous 2 weeks, or a systolic BP ≥140 mm Hg, or a diastolic BP ≥90 mm Hg (BP ≥140/90 mm Hg) [20]. The threshold of BP ≥130/80 mm Hg was also used as an alternative definition for hypertension in this study [3].

### Statistical Analysis

Appropriate weights were applied in the analyses to represent the overall Chinese adult population aged ≥18 years according to the multistage probability sampling design of the survey. Weight coefficients were calculated based on the 2010 China population census data and the sampling scheme of the current survey [21]. The final weight was the product of sampling weight, nonresponse weight, and poststratification weight for each participant (Supplementary Methods in Multimedia Appendix 1). The distribution of weight coefficients was provided in Figure S1 in Multimedia Appendix 1. Characteristics of the study population were presented in men and women, separately with continuous variables in means (95% CIs) and categorical variables in percentages (95% CIs).

We first tested for multiplicative interactions between SES indicators and sex by adding interaction terms to regression models in association with BP levels using a likelihood-ratio test. If there was a significant interaction, results were to be presented in stratified subgroups. BP characteristics including systolic and diastolic BP levels, and prevalence of hypertension in Chinese men and women were described by SES categories.

We used unadjusted and multivariable-adjusted linear regression models to examine the associations of sex with systolic and diastolic BP levels stratified by SES indicators. Because the prevalence of hypertension was not rare in the study participants (>10%), prevalence ratio (PR), a better approximation to the risk ratio than the odds ratio, was calculated using unadjusted and multivariable-adjusted Cox regression models with robust variance and a constant as the time variable to assess the association between sex and prevalent hypertension stratified by SES indicators [22,23]. Confounders adjusted in the multivariable models were based on knowledge of their associations with sex and BP characteristics and included age, smoking status, drinking status, physical activity, diet score, obesity, taking antihypertensive medications (in linear regression models), and other SES indicators.

BP characteristics in Chinese men and women were examined further in subcategories with cross-tabulation of education and another SES indicator because education is proven to be the most important SES indicator related to health. In addition, because BP levels are highly dependent on age [24], the study population was further categorized into young adults with age 18‐44 years, middle-aged adults with age 45‐64 years, and older adults with age ≥65 years. Differences in BP characteristics between men and women, and the associations between sex and BP levels as well as hypertension prevalence were examined in these subcategories. In addition, the associations between the composite SES score and BP characteristics were examined in men and women, separately using the overall low-SES group as the reference. Women-to-men relative ratio was obtained by comparing the PRs from men and women in each stratified group of the composite SES score to assess the effects of sex difference.

Finally, we calculated the number needed to be exposed for one additional person to benefit (NNEB) from increasing the education duration in men and women, separately using adjusted PR to indicate the impact of improving SES on hypertension prevention [25].

Imputations were performed using a fully conditional specification method for variables with missing values exceeding 5%, which included the healthy diet score (missing 6.2%) and the annual household income (missing 14.7%). We tested the assumption of missingness at random by including missingness as a variable and estimating its associations with variables with full information and found convincing evidence supporting missingness at random assumption. Missing values for other variables were not imputed because missingness was <1.5% for these variables.

All the data analyses were performed using R (version 4.2.2; R Foundation for Statistical Computing). Statistical significance was defined as a 2-sided *P* value less than .05.

## Results

General characteristics of study participants stratified by sex are shown in Table 1. Men were more likely to have a longer duration of education, a higher annual household income, and higher proportions of an overall high SES, current smokers, and current drinkers than women. Systolic and diastolic BP levels, and prevalence of hypertension were significantly higher in men than women (all *P* values <.001). Stratified by sex and each SES indicator, similar trends are shown in Tables S1-S5 in Multimedia Appendix 1. Because significant multiplicative interactions between all included SES indicators and sex in associations with BP characteristics were found, subsequent analyses were stratified on them (*P*_interaction_<.05).

**Table 1.** Baseline characteristics of participants stratified by sex. Data are weighted means or percentages (95% CI). Comparisons between men and women were conducted using ANOVA for continuous variables and chi-square tests for categorical variables.

Characteristics	Men	Women	*P* value
Number of participants	45,143	53,515	—^a^
Age (in years), mean (95% CI)	42.8 (42.1-43.6)	43.6 (42.7-44.4)	.001
**Age category (in years), percentage (95% CI)**			<.001
18‐44	58.1 (56.1-60.1)	56.8 (54.6-59.1)	
45‐64	31.9 (30.4-33.5)	31.8 (30.2-33.5)	
≥65	10.0 (9.1-10.8)	11.3 (10.3-12.3)	
**Education (%), percentage (95% CI)**			<.001
Duration of education<6 years	12.6 (11.2-13.9)	26.6 (23.8-29.4)	
Duration of education 6‐9 years	57.7 (55.0-60.3)	50.2 (47.6-52.9)	
Duration of education>9 years	30.0 (26.7-32.8)	23.1 (19.9-26.3)	
**Economic development (%), percentage (95% CI)**			.45
Underdeveloped	33.3 (24.0-42.5)	33.9 (24.5-43.3)	
Intermediately developed	33.9 (24.9-43.0)	33.1 (24.2-41.9)	
Developed	32.8 (24.2-41.4)	33.1 (24.4-41.7)	
Living in urban area (%), percentage (95% CI)	31.1 (23.0-39.2)	31.4 (23.1-39.6)	.77
Annual household income (10,000 Chinese Yuan [US $1477.2]), mean (95% CI)	2.75 (2.51-2.99)	2.62 (2.39-2.86)	.03
Living in the northern China (%), percentage (95% CI)	48.3 (38.8-57.8)	49.0 (39.4-58.5)	.40
**Composite SES**^**b**^ **score, (%), percentage (95% CI)**			<.001
Low SES (0-1)	23.2 (17.8-28.6)	29.7 (23.9-35.5)	
Intermediate SES (2-3)	50.3 (44.9-55.7)	46.6 (41.8-51.4)	
High SES (4-5)	26.5 (20.0-33.0)	23.7 (17.7-29.7)	
Current smoking (%), percentage (95% CI)^c^	53.3 (51.3-55.3)	2.5 (1.9-3.0)	<.001
Current drinking (%), percentage (95% CI)	50.3 (47.8-52.7)	8.1 (7.0-9.2)	<.001
Physical activity (METs-h/week), mean (95% CI)^d^	94.6 (87.3-102.0)	79.8 (73.3-86.2)	<.001
**Healthy diet score (%), percentage (95% CI)**			.10
0‐1 points	36.9 (33.7-40.0)	36.1 (32.9-39.3)	
2‐3 points	61.6 (58.7-64.6)	62.2 (59.3-65.2)	
4‐5 points	1.5 (1.0-2.0)	1.7 (1.1-2.3)	
BMI (kg/m^2^), mean (95% CI)^c^	23.8 (23.6-23.9)	23.7 (23.5-23.9)	.17
Waist circumference (cm), mean (95% CI)^c^	82.1 (81.5-82.8)	78.3 (77.7-78.8)	<.001
Obesity (%), percentage (95% CI)^c^	11.9 (10.8-12.9)	12.1 (11.1-13.2)	.51
Systolic BP^e^ (mm Hg), mean (95% CI)^c^	133.3 (132.4-134.3)	130.0 (128.7-131.3)	<.001
Diastolic BP^e^ (mm Hg), mean (95% CI)^c^	81.6 (81.0-82.2)	80.0 (79.4-80.6)	<.001
Prevalence of hypertension (%), percentage (95% CI)^c^^f^	63.7 (61.4-66.0)	55.0 (52.6-57.4)	<.001
Prevalence of hypertension (%), percentage (95% CI)^c^^g^	35.3 (33.2-37.3)	32.0 (30.0-34.0)	<.001

a Not applicable.

b SES: socioeconomic status.

c There were 4 missing values for current smoking, 85 missing values for BMI, 75 missing values for waist circumference, 85 missing values for obesity, 67 missing values for systolic BP, 66 missing values for diastolic BP, and 62 missing values for status of hypertension.

d MET: metabolic equivalent.

e BP: blood pressure.

f Hypertension was defined as a self-report of using antihypertensive medications within the previous 2 weeks, or a systolic BP ≥130 mm Hg, or a diastolic BP ≥80 mm Hg.

g Hypertension was defined as a self-report of using antihypertensive medications within the previous 2 weeks, or a systolic BP ≥140 mm Hg, or a diastolic BP ≥90 mm Hg.

Table 2 shows the distribution of BP characteristics in Chinese men and women stratified by SES categories. Systolic and diastolic BP levels, and prevalence of hypertension were generally higher in men than women in each group stratified by SES indicators. Using men as the reference, the associations between sex and BP characteristics in different categories of SES were examined both in unadjusted (Table S6 in Multimedia Appendix 1) and multivariable-adjusted models (Table 3). There were no significant sex differences in BP characteristics in participants with duration of education<6 years. However, women were associated with significantly lower BP levels and hypertension prevalence compared with men in SES categories with longer durations of education. For example, in participants with >9 years of education, women were associated with a significantly lower level of systolic BP (β coefficient=−7.93, 95% CI −8.83 to −7.04) and diastolic BP (β coefficient=−3.28, 95% CI −3.82 to −2.75), and 37% lower probabilities of having prevalent hypertension defined by BP ≥140/90 mm Hg (PR 0.63, 95% CI 0.58-0.68) compared with men. Similar trends were found in groups stratified by other SES indicators with more evident sex differences in BP characteristics in participants having more favorable SES (Table 3). Similar results were found in hypertension defined by BP ≥130/80 mm Hg (Table S7 in Multimedia Appendix 1).

**Table 2.** Distribution of BP characteristics in Chinese men and women stratified by socioeconomic status (SES) categories. Data are weighted means or percentages (95% CI).

	Systolic BP^a^ (mm Hg), mean (95% CI)		Diastolic BP^a^ (mm Hg), mean (95% CI)		Prevalence of hypertension^b^ (%), percentage (95% CI)	
	Men	Women	Men	Women	Men	Women
**Education**						
Duration of education<6 years	140.6 (139.0-142.2)	141.4 (139.5-143.4)	82.5 (81.7-83.2)	83.0 (82.3-83.7)	48.7 (45.5-52.0)^c^	51.7 (48.3-55.1)^c^
Duration of education 6‐9 years	133.2 (132.1-134.3)^c^	128.1 (126.8-129.5)^c^	81.4 (80.8-82.1)^c^	79.8 (79.2-80.4)^c^	34.7 (32.4-37.1)^c^	28.2 (26.0-30.4)^c^
Duration of education>9 years	130.6 (129.5-131.7)^c^	120.9 (119.4-122.4)^c^	81.6 (81.0-82.3)^c^	77.1 (76.4-77.8)^c^	30.5 (28.1-32.9)^c^	17.6 (15.7-19.6)^c^
**Economic development**						
Underdeveloped	131.5 (129.8-133.2)^c^	128.9 (126.3-131.5)^c^	79.8 (78.9-80.6)^c^	78.9 (77.9-79.9)^c^	30.1 (26.8-33.5)	29.0 (25.2-32.8)
Intermediately developed	133.3 (131.7-134.9)^c^	130.2 (128.0-132.5)^c^	81.9 (80.9-82.8)^c^	80.2 (79.3-81.2)^c^	35.0 (31.8-38.2)^c^	32.3 (28.7-35.8)^c^
Developed	135.2 (133.8-136.7)^c^	130.9 (129.0-132.8)^c^	83.2 (82.5-83.9)^c^	80.9 (80.2-81.6)^c^	40.7 (37.9-43.4)^c^	34.8 (32.1-37.6)^c^
**Area**						
Urban	133.7 (132.3-135.1)^c^	128.8 (127.0-130.6)^c^	82.4 (81.7-83.1)^c^	79.7 (79.0-80.5)^c^	37.7 (34.7-40.7)^c^	31.7 (29.0-34.5)^c^
Rural	133.2 (131.9-134.5)^c^	130.6 (128.8-132.3)^c^	81.2 (80.5-82.0)^c^	80.1 (79.4-80.9)^c^	34.1 (31.5-36.7)^c^	32.1 (29.4-34.8)^c^
**Annual household Income**						
Higher income	131.9 (130.9-133.0)^c^	126.4 (125.1-127.6)^c^	81.7 (81.1-82.3)^c^	79.1 (78.6-79.6)^c^	33.3 (31.0-35.5)^c^	26.2 (24.4-28.0)^c^
Lower income	134.9 (133.7-136.2)^c^	133.8 (132.1-135.4)^c^	81.5 (80.7-82.2)^c^	81.0 (80.2-81.7)^c^	37.6 (34.9-40.2)	37.9 (35.1-40.8)
**Region**						
South	130.8 (129.5-132.1)^c^	126.4 (124.6-128.2)^c^	80.5 (79.7-81.2)^c^	78.5 (77.7-79.2)^c^	30.2 (27.6-32.8)^c^	26.6 (23.8-29.4)^c^
North	136.1 (135.0-137.2)^c^	133.7 (132.4-135.0)^c^	82.8 (82.1-83.6)^c^	81.6 (81.1-82.1)^c^	40.7 (38.3-43.1)^c^	37.6 (35.5-39.8)^c^

a BP: blood pressure.

b Hypertension was defined as a self-report of using antihypertensive medications within the previous 2 weeks, or a systolic BP ≥140 mm Hg, or a diastolic BP ≥90 mm Hg.

c *P*<.05 comparing BP characteristics between men and women.

**Table 3.** The association between sex and blood pressure (BP) characteristics in different categories of socioeconomic status (SES) in multivariable-adjusted models. β coefficients (95% CIs) or prevalence ratios (95% CIs) for women versus men are presented. The model was adjusted for age, smoking status, drinking status, physical activity, diet score, obesity, taking antihypertensive medications (in models for systolic blood pressure [BP] and diastolic BP), and other SES indicators in the table.

	Systolic BP		Diastolic BP		Hypertension^a^	
	β coefficient (95% CI)	*P*_interaction_	β coefficient (95% CI)	*P*_interaction_	PR^b^ (95% CI)	*P*_interaction_
**Education**		<.001		<.001		<.001
Duration of education<6 years	–0.70 (–1.76 to 0.37)		–0.55 (–1.19 to 0.09)		1.03 (0.97 to 1.10)	
Duration of education 6‐9 years	–4.08 (–4.85 to –3.31)		–1.13 (–1.68 to –0.58)		0.84 (0.79 to 0.90)	
Duration of education>9 years	–7.93 (–8.83 to –7.04)		–3.28 (–3.82 to –2.75)		0.63 (0.58, 0.68)	
**Economic development**		.045		.001		.01
Underdeveloped	–3.85 (–5.07 to –2.63)		–0.86 (–1.80 to 0.09)		0.88 (0.80 to 0.98)	
Intermediately developed	–4.83 (–5.94 to –3.72)		–1.83 (–2.50 to –1.15)		0.85 (0.76 to 0.94)	
Developed	–5.10 (–6.03 to –4.16)		–2.25 (–2.71 to –1.79)		0.79 (0.75 to 0.84)	
**Area**		.002		<.001		.003
Urban	–6.06 (–7.08 to –5.04)		–2.60 (–3.04 to –2.15)		0.76 (0.72 to 0.80)	
Rural	–4.00 (–4.79 to –3.21)		–1.23 (–1.81 to –0.64)		0.87 (0.81 to 0.94)	
**Annual household income**		<.001		<.001		<.001
Higher income	–6.13 (–6.83 to –5.44)		–2.36 (–2.76 to –1.96)		0.75 (0.71 to 0.79)	
Lower income	–3.13 (–3.99 to –2.27)		–0.94 (–1.60 to –0.29)		0.92 (0.85 to 0.99)	
**Region**		<.001		.005		.07
South	–5.83 (–6.61 to –5.05)		–2.25 (–2.68 to –1.81)		0.78 (0.72 to 0.83)	
North	–3.57 (–4.45 to –2.69)		–1.14 (–1.86 to –0.43)		0.87 (0.81 to 0.93)	

a Hypertension was defined as a self-report of using antihypertensive medications within the previous 2 weeks, or a systolic BP ≥140 mm Hg, or a diastolic BP ≥90 mm Hg.

b PR: prevalence ratio.

**Figure 1.**
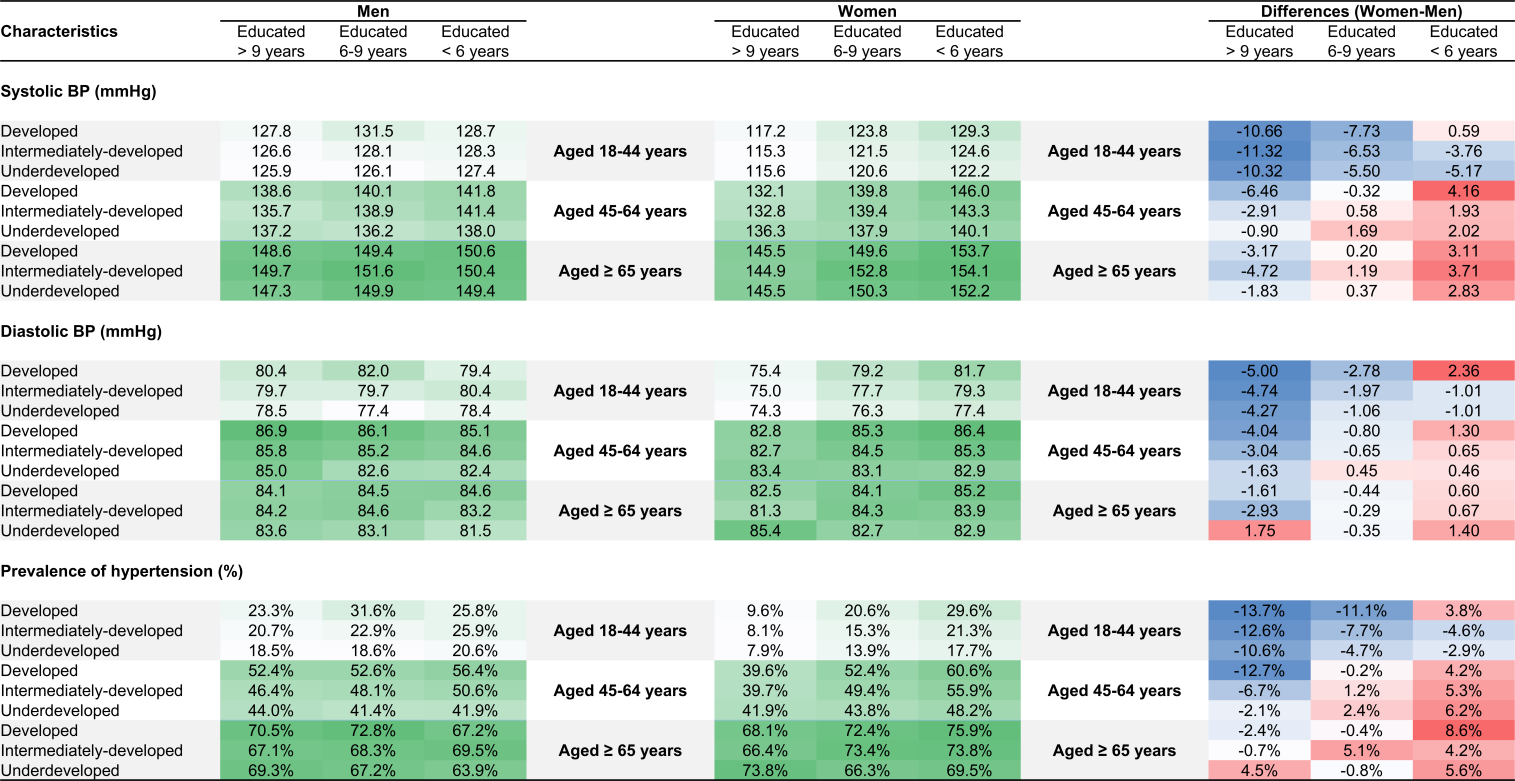
Distribution of BP characteristics in subcategories of education duration and economic development status stratified by age groups in men and women (hypertension was defined as a self-report of using antihypertensive medications within the previous 2 weeks, or a systolic BP ≥140 mm Hg, or a diastolic BP ≥90 mm Hg). Data are weighted means or percentages. The changing green color depicts different levels of BP and hypertension prevalence with darker green indicating higher levels. The changing blue and red colors depict differences between women and men with darker blue indicating lower levels in women versus men and darker red indicating higher levels in women versus men. BP: blood pressure.

Mean systolic BP levels, mean diastolic BP levels, and prevalence of hypertension in Chinese men and women as well as the difference between Chinese men and women in subcategories with cross-tabulation of education and another SES indicator are shown in Figure 1 and Figures S2-S5 in Multimedia Appendix 1. By labeling with shades of colors, a clear pattern of sex differences in BP characteristics was seen. In participants with longer duration of education and more developed economic status, women had lower BP levels and hypertension prevalence than men, while in participants with less education and less developed economic status, women tended to have higher BP levels and hypertension prevalence than men. The same pattern was seen for multivariable-adjusted β coefficients and PRs demonstrating associations between sex and BP characteristics (Figures S6-S9 in Multimedia Appendix 1).

**Figure 2.**
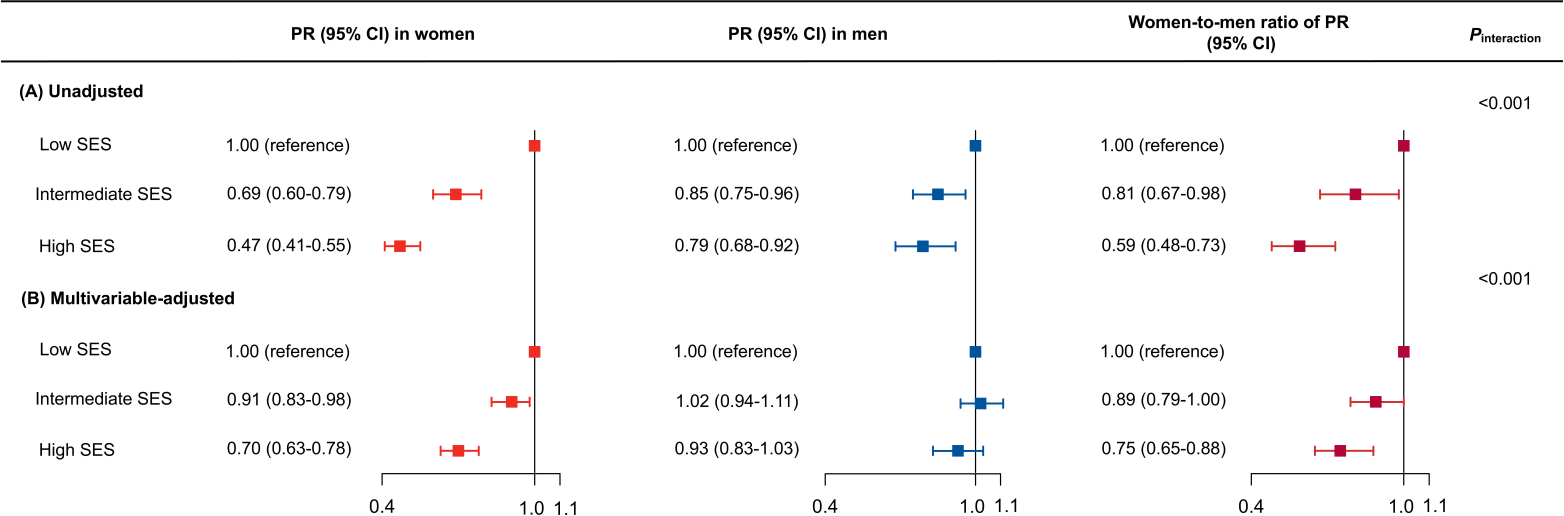
Associations between the overall SES and prevalent hypertension defined by BP ≥140/90 mm Hg in Chinese men and women in unadjusted (**A**) and multivariable-adjusted (**B**) models (hypertension was defined as a self-report of using antihypertensive medications within the previous 2 weeks, or a systolic BP ≥140 mm Hg, or a diastolic BP ≥90 mm Hg). The low-SES group was the reference group. Data are shown as PR (95% CIs). The multivariable model included age, smoking status, drinking status, physical activity, diet score, and obesity. BP: blood pressure; PR: prevalence ratio; SES: socioeconomic status.

Figure S10 in Multimedia Appendix 1 showed the percentages of different composite SES scores in men and women. Women tended to have higher percentages of low SES scores and lower percentages of high SES scores than men. Using the overall low-SES group as the reference, a 9% lower probability (PR 0.91, 95% CI 0.83-0.98) and a 30% lower probability (PR 0.70, 95% CI 0.63-0.78) of having hypertension defined by BP ≥140/90 mm Hg were found in women with an overall intermediate SES and high SES, respectively after multivariable adjustment (Figure 2). In contrast, no significant reduction in the probability of having hypertension was observed in the intermediate or the high-SES group versus the low-SES group in men. Women-to-men ratios of PRs showed that there were significant differences between men and women in the reduced probabilities of having hypertension in the intermediate-SES group or the high-SES group versus the low-SES group (Figure 2). Similar trends were found in hypertension defined by BP ≥130/80 mm Hg (Figure S11 in Multimedia Appendix 1).

NNEB analyses showed that increasing every 13 men of their durations of education from <6 years to >9 years could avoid one case of hypertension (if defined by BP ≥140/90 mm Hg). In contrast, only 4 women were needed to increase their durations of education from <6 years to >9 years to avoid one case of hypertension.

## Discussion

Using a nationally representative sample of 98,658 Chinese adults across mainland China, we found that there were significant sex differences in BP levels and hypertension prevalence, and SES had a significant influence on these sex differences. Women had an increasingly favorable BP level and prevalent hypertension versus men with an improving SES. NNEB analyses indicated that a much smaller number of women are needed to be educated to avoid one case of hypertension than men. Our findings provided evidence of the potential effects of SES on sex differences regarding hypertension, and suggested that targeted public health policies in men and women are needed and interventions to alleviate the socioeconomic inequities, especially in women are warranted to reduce the hypertension burden.

Sex differences in prevalent hypertension found in our study were consistent with results from most but not all previous studies [4,26,27]. A pooled analysis of 135 population-based studies from 90 countries revealed global disparities in hypertension prevalence in men and women, and that the overall prevalence of hypertension was almost identical in men (n=520.1 millions, 31.7%) and women (n=518.8 millions, 31.2%) in low- and middle-income countries though still higher in men than in women (n=174.2 millions, 31.6% vs n=174.7 millions, 25.3%) in high-income countries [28]. These numbers further highlight the sex- and region-specific differences in hypertension prevalence. Previous studies suggested that a higher prevalence of hypertension in men than women might result from the androgen-specific effects of increased BP through the renin-angiotensin system or the potentially protective effects of estrogen on the vasculature and the sympathetic nervous system [29]. Unhealthy lifestyle behaviors including smoking and drinking as revealed in this study and higher energy, fat, and sugar intakes in men, which were associated with an increased risk of hypertension [30-33], might also count.

Using single, cross-tabulated, and composite SES, our study indicated that SES played an important role in the sex differences of hypertension, and women with an improving SES were significantly associated with lower BP levels and hypertension prevalence, which is partly consistent with previous studies [34-37]. A cross-sectional population-based study in France showed that education and income inequalities were stronger among women than among men [34]. However, the China Kadoorie, Biobank study showed no obvious sex difference in the association between hypertension and income in different GDP per capita areas [36]. Another large cohort study of Chinese population demonstrated an inverse association between SES and hypertension observed only in women [37]. However, sex differences were not compared directly in these studies [34-37] and often single SES indicators were included without considering regional disparities in China.

It has been recognized that SES indicators are associated with health outcomes partly through their impacts on lifestyle behaviors [38]. Previous studies have demonstrated that lower SES was associated with unhealthy lifestyle behaviors including lower physical activity, lower fresh food intake, and higher sodium intake [39]. In this study, the proportions of current smoking and excess drinking decreased while the leisure-time physical activity and diet score improved with increasing education levels in both men and women (Table S1 in Multimedia Appendix 1). It is worth noting that with increasing durations of education, levels of BMI and waist circumference increased in men but decreased in women. This was also found in previous studies which demonstrated that higher education levels were more likely to be associated with overweight or obesity in men while this association was reversed in women [40]. This could partly contribute to the widened differences in BP levels between women and men with increasing durations of education found in this study.

Participants with more favorable SES such as those living in the urban area or living in the developed economic region had higher levels of leisure-time physical activity and diet scores though at the same time higher BMI in both men and women (Tables S2 and S3 in Multimedia Appendix 1). Previous studies have elucidated that the protective impact of leisure-time physical activity on hypertension tended to be more evident in women, especially in those with overweight or obesity [41]. Dietary intervention also lowered systolic BP more in women than in men, which might lead to more significant sex differences in participants with favorable SES status [42]. Consistent with previous studies [43,44], we found that participants living in the north were more likely to have higher BMI and waist circumference than participants living in the south in both men and women (Table S5 in Multimedia Appendix 1). Emerging evidence showed that there was a stronger association between obesity or BMI and hypertension in women than in men, which might potentially account for the less significant sex difference in the north than in the south in this study [45,46]. However, the potential mechanisms of SES affecting sex differences in hypertension warrant further investigation.

The major strength of this study was the large study sample recruited from 162 study sites located across mainland China and representative of Chinese adults aged ≥18 years with a wide coverage of SES evaluated using 5 SES indicators. There are also several limitations. First, BP levels were measured at a single point in time, although standard procedures were followed and 3 measurements were conducted for each participant. Second, information on some SES indicators was self-reported and this may lead to misclassifications such as for annual household income. Third, causality cannot be inferred due to the cross-sectional nature of this study. However, sex is a born attribute and SES indicators were generally determined at early stages of life. The temporality of their associations with BP or hypertension is somehow clear. Finally, the generalizability of our findings to other populations in countries with different levels of development might be limited.

In conclusion, we found significant sex differences in BP levels and hypertension prevalence in Chinese men and women, and SES played an important part in the sex disparities. Women with favorable SES were inversely associated with BP levels and prevalent hypertension compared to men. Policies at increasing SES in women such as fostering longer durations of education to alleviate socioeconomic inequalities can be both effective and efficient toward a reduced burden of hypertension. Future studies are needed to illustrate the interplay of biological, social, and behavioral factors in sex differences in health outcomes.

## Supplementary material

10.2196/63144 Multimedia Appendix 1 Additional information.

## Abbreviations

BP: blood pressure
CDC: Center for Disease Control and Prevention
CNY: Chinese Yuan
GDP: gross domestic product
NNEB: number needed to be exposed for one additional person to benefit
PR: prevalence ratio
SES: socioeconomic status
