# Supplementary material for: Sex Difference and Socioeconomic Inequity in Chinese People With Hypertension: National Cross-Sectional Survey Study
Source: JMIR Public Health Surveill. 2024 Nov 20;10:e63144. doi: 10.2196/63144 (PMC11611794; doi:10.2196/63144)
Supplement: Multimedia Appendix 1 [file publichealth-v10-e63144-s001.docx]

| **Table of contents** | |
| --- | --- |
| **Supplementary methods** | Page 3-5 |
| **Figure S1.** Distribution of weight coefficients | Page 6 |
| **Figure S2.** Distribution of BP characteristics in subcategories of education duration and economic development status stratified by age groups in men and women ^a^ | Page 7 |
| **Figure S3.** Distribution of BP characteristics in subcategories of education duration and area stratified by age groups in men and women | Page 8 |
| **Figure S4.** Distribution of BP characteristics in subcategories of education duration and annual household income stratified by age groups in men and women | Page 9 |
| **Figure S5.** Distribution of BP characteristics in subcategories of education duration and region stratified by age groups in men and women | Page 10 |
| **Figure S6.** Associations between sex and BP characteristics in subcategories of education duration and economic development status stratified by age groups after multivariable adjustment | Page 11 |
| **Figure S7.** Association between sex and BP characteristics in subcategories of education duration and area stratified by age groups after multivariable adjustment | Page 12 |
| **Figure S8.** Association between sex and BP characteristics in subcategories of education duration and annual household income stratified by age groups after multivariable adjustment | Page 13 |
| **Figure S9.** Association between sex and BP characteristics in subcategories of education duration and region stratified by age groups after multivariable adjustment | Page 14 |
| **Figure S10.** Percentages of composite SES scores in Chinese men and women | Page 15 |
| **Figure S11.** Associations between the overall SES and prevalent hypertension defined by BP ≥130/80 mmHg in Chinese men and women in unadjusted (A) and multivariable-adjusted (B) models ^a^ | Page 16 |
| **Table S1.** Baseline characteristics of participants stratified by sex and education | Page 17 |
| **Table S2.** Baseline characteristics of participants stratified by sex and economic development status | Page 18 |
| **Table S3.** Baseline characteristics of participants stratified by sex and area | Page 19 |
| **Table S4.** Baseline characteristics of participants stratified by sex and annual household income | Page 20 |
| **Table S5.** Baseline characteristics of participants stratified by sex and region | Page 21 |
| **Table S6**. The association between sex and BP characteristics in different categories of SES in unadjusted models | Page 22 |
| **Table S7**. Prevalence of hypertension and the association between sex and hypertension defined by BP ≥130/80 mmHg in different categories of SES in unadjusted and multivariable-adjusted models | Page 23 |

**Supplementary methods**

**Multistage** **probability sampling design and weighting methods**

The China Noncommunicable Disease Surveillance Study was designed to select a nationally representative sample of the general population, covering major geographic areas of all 31 provinces, autonomous regions, and municipalities in mainland China. The study recruited participants from 162 study sites within the National Disease Surveillance Point System of Chinese Center for Disease Control and Prevention (China CDC). At each site, a complex, multistage, probability sampling design was used to select participants who were representative of civilian, noninstitutionalized Chinese adults.

All the participants were weighted in the sequence of sampling weight, non-response weight, and post-stratification weight. The final weight of each participant was the product of the sampling weight, the non-response weight, and the post-stratification weight.

**1. Calculation of sampling weight**

Let *i* be an individual sampling participant. According to the survey design, the following equations were used to calculate the sampling weights.

**a.** Let *w_si1_* be the sampling weight for counties in rural areas or districts in urban areas. It is the reciprocal of the sampling probability of county/district based on the stratified simple random sampling:

*w_si1_* = $\frac{Total number of counties/districts in the stratum where individual i belongs}{Number of sampled counties/districts in the stratum where individual i belongs}$

**b.** Let *w_si2_* be the sampling weight for townships in rural areas or subdistricts in urban areas. It is the reciprocal of the sampling probability of township/subdistrict based on the probability proportional to size (PPS) sampling:

*w_si2_* = $\frac{Population size of the study site where individual i belongs (Popi1)}{4 ⅹ population size of the township/subdistrict where i belongs (Popi2)}$

**c.** Let *w_si3_* be the sampling weight for administrative villages in rural areas or neighborhood communities in urban areas. It is the reciprocal of the sampling probability of village/community based on the PPS sampling:

*w_si3_* = $\frac{Population size of the township/subdistrict where individual i belongs (Popi2)}{3 ⅹ population size of the village/community where individual i belongs (Popi3)}$

**d.** Let *w_si4_* be the sampling weight for individuals. It is the reciprocal of the sampling probability of an individual:

*w_si4_* = $\frac{Number of people \geq18 years in the village/community where individual i belongs (Popi3)}{Number of sampled individuals in the village/community where individual i belongs (Popi4)}$

Therefore, the sampling weight for each participant (*w_si_*) is:

*w_si_ = w_si1_* ⅹ *w_si2_* ⅹ *w_si3_* ⅹ *w_si4_* ⅹ *percentage of people aged ≥18 years in China*

**2. Calculation of** **non-response weight**

If there are participants who have not responded because they refuse or are not available, non-response weight should be assigned to those who have responded at each stratum. Suppose that the non-response was at random, the non-response weight is calculated as:

*w_nr_* = $\frac{Planned sample size at each stratum}{Number of respondents at each stratum}$

**3. Calculation of post-stratification weight**

Post-stratification weight was assigned to adjust the deviation in important variables of the sampled population from the total Chinese population aged ≥18 years.

1. Definition of sample and population

Sample: sampled participants weighted by sampling weight and non-response weight

Population: Chinese population aged ≥18 years based on the 2010 China population census data

**b.** There were a total of 156 strata based on stratification variables including sex, age groups, urban or rural areas, and geographical regions.

Table. Stratification variables and number of strata

| **Stratification variable** | **Number of strata** | **Description** |
| --- | --- | --- |
| Sex | 2 | Men, women |
| Age groups | 13 | 18-24, 25-29, 30-34, 35-39,  40-44, 45-49, 50-54, 55-59,  60-64, 65-69, 70-74, 75-79, 80+ |
| Urban or rural areas | 2 | Urban, rural |
| Geographical regions | 3 | East, middle, west |

**c.** Calculation of post-stratification weight

*w_pk_* = $\frac{Population size at stratum k}{Weighted number of participants at stratum k}$

Weight in the above calculation is the product of sampling weight and non-response weight.

**4. The final weight**

The final weight for each participating individual (individual *i* at stratum *k*) is the product of sampling weight, non-response weight, and post-stratification weight.

$w_{final}=w_{si}\times w_{nr}\times w_{pk}$

**
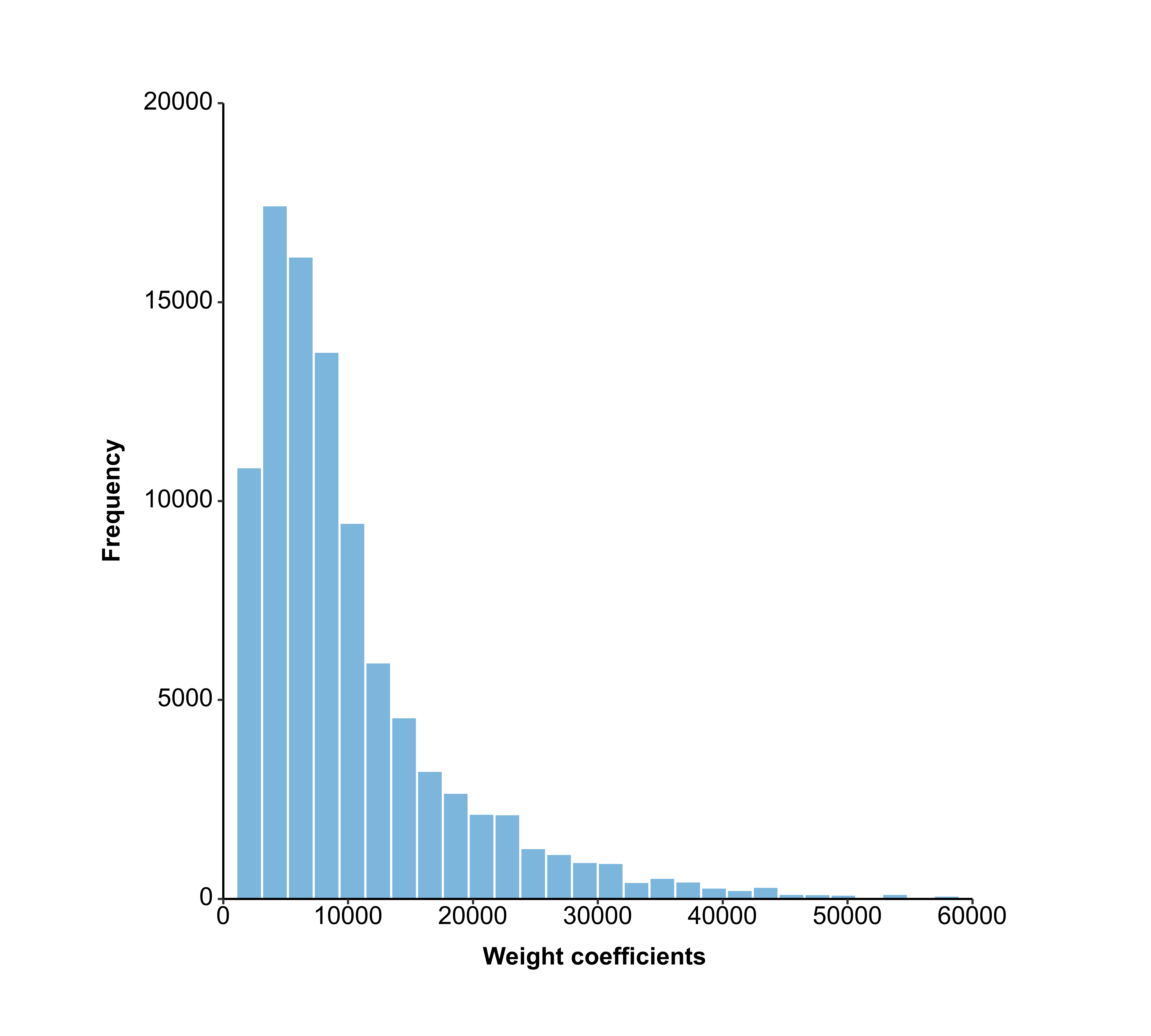
Figure S1.** Distribution of weight coefficients

**
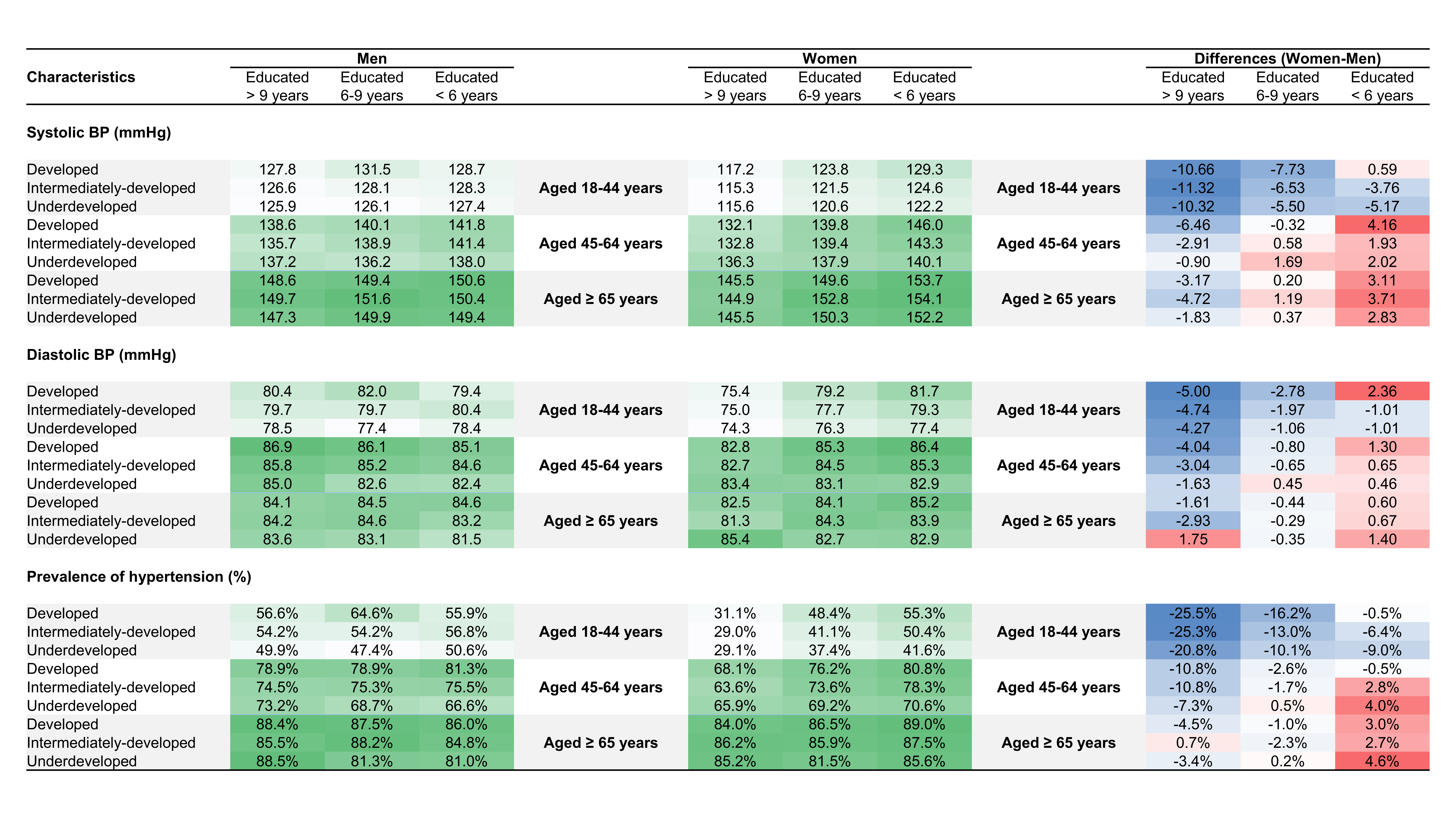
Figure S2.** Distribution of BP characteristics in subcategories of education duration and economic development status stratified by age groups in men and women ^a^

Data are weighted means or percentages. The changing green color depicts different levels of BP and hypertension prevalence with darker green indicating higher levels. The changing blue and red colors depict differences between women and men with darker blue indicating lower levels in women *vs.* men and darker red indicating higher levels in women *vs.* men.

^a^ Hypertension was defined as a self-report of using anti-hypertensive medications within the previous 2 weeks, or a systolic BP ≥130 mmHg, or a diastolic BP ≥80 mmHg.

Abbreviation: BP, blood pressure.

**
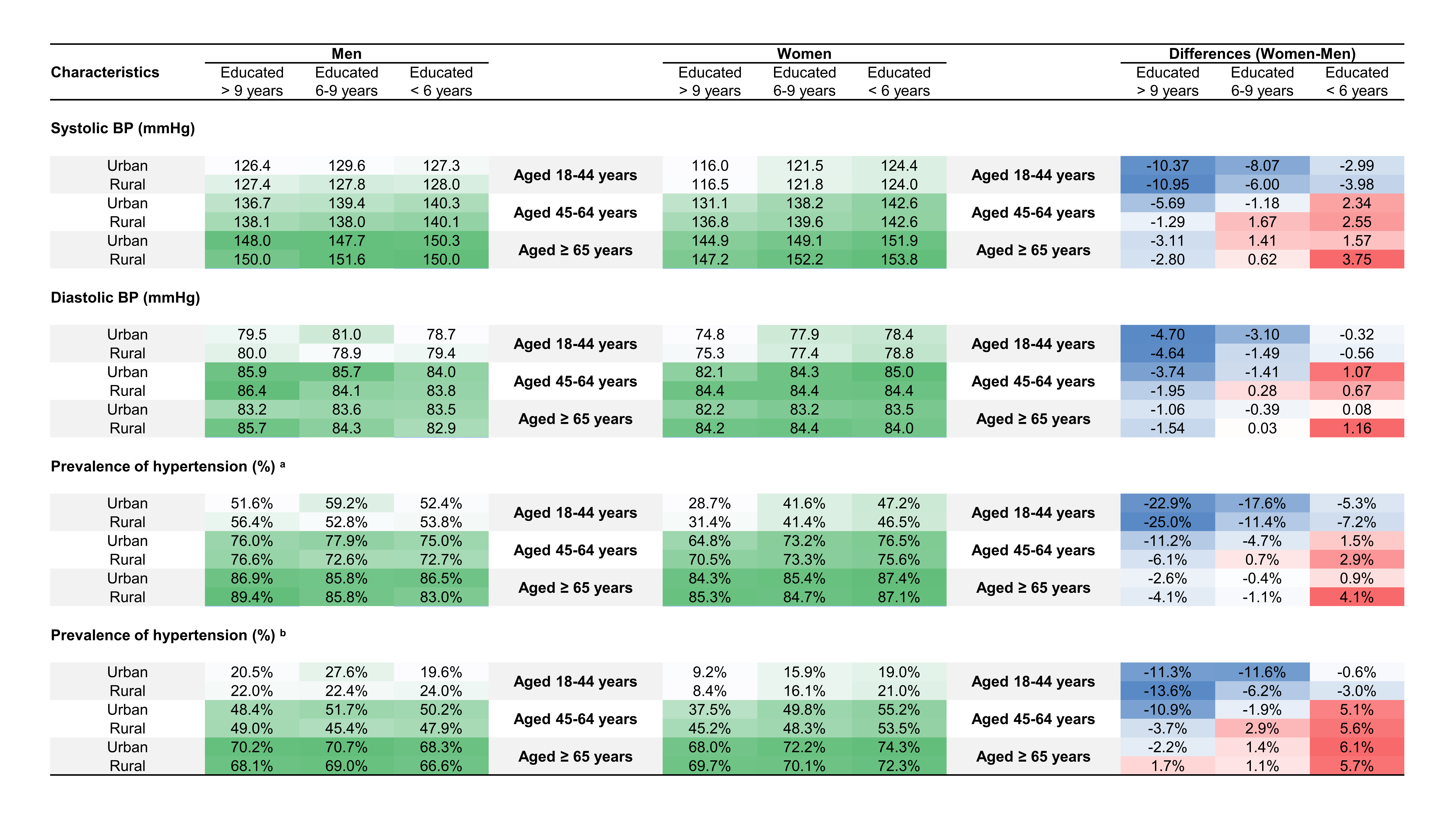
Figure S3.** Distribution of BP characteristics in subcategories of education duration and area stratified by age groups in men and women

Data are weighted means or percentages. The changing green color depicts different levels of BP and hypertension prevalence with darker green indicating higher levels. The changing blue and red colors depict differences between women and men with darker blue indicating lower levels in women *vs.* men and darker red indicating higher levels in women *vs.* men.

^a^ Hypertension was defined as a self-report of using anti-hypertensive medications within the previous 2 weeks, or a systolic BP ≥130 mmHg, or a diastolic BP ≥80 mmHg.

^b^ Hypertension was defined as a self-report of using anti-hypertensive medications within the previous 2 weeks, or a systolic BP ≥140 mmHg, or a diastolic BP ≥90 mmHg.

Abbreviation: BP, blood pressure.

**
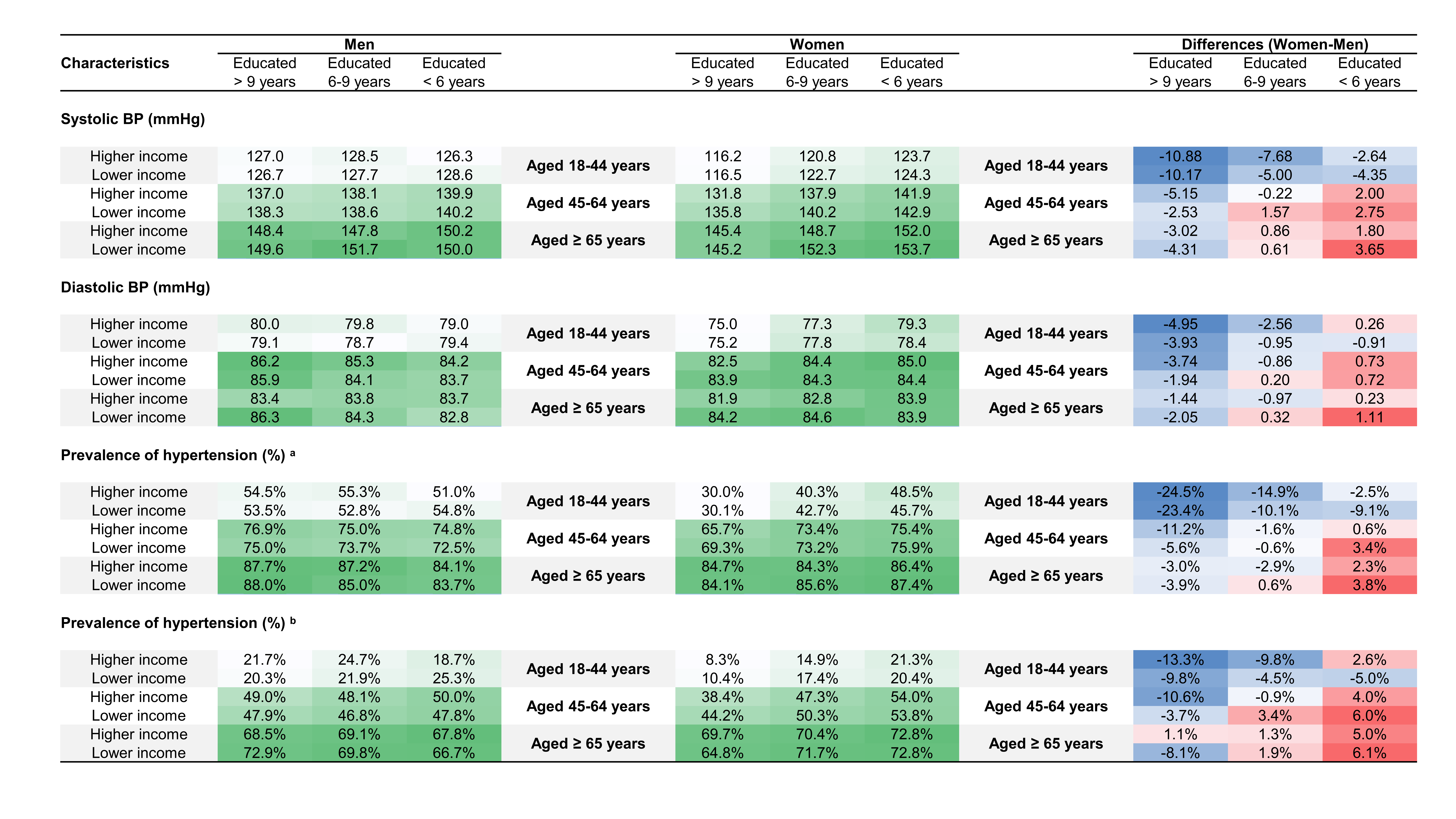
Figure S4.** Distribution of BP characteristics in subcategories of education duration and annual household income stratified by age groups in men and women

Data are weighted means or percentages. The changing green color depicts different levels of BP and hypertension prevalence with darker green indicating higher levels. The changing blue and red colors depict differences between women and men with darker blue indicating lower levels in women *vs.* men and darker red indicating higher levels in women *vs.* men.

^a^ Hypertension was defined as a self-report of using anti-hypertensive medications within the previous 2 weeks, or a systolic BP ≥130 mmHg, or a diastolic BP ≥80 mmHg.

^b^ Hypertension was defined as a self-report of using anti-hypertensive medications within the previous 2 weeks, or a systolic BP ≥140 mmHg, or a diastolic BP ≥90 mmHg.

Abbreviation: BP, blood pressure.

**
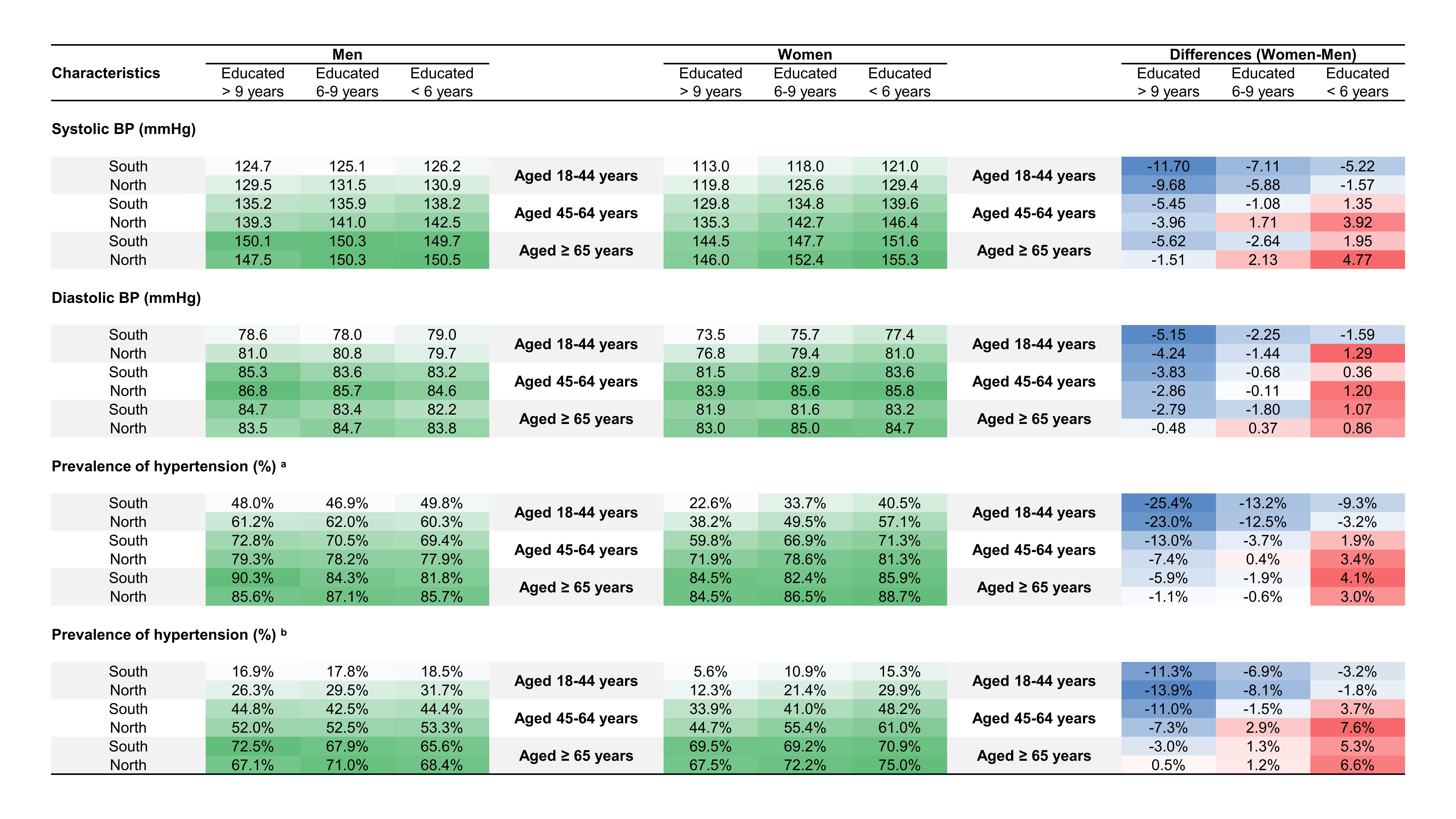
Figure S5.** Distribution of BP characteristics in subcategories of education duration and region stratified by age groups in men and women

Data are weighted means or percentages. The changing green color depicts different levels of BP and hypertension prevalence with darker green indicating higher levels. The changing blue and red colors depict differences between women and men with darker blue indicating lower levels in women *vs.* men and darker red indicating higher levels in women *vs.* men.

^a^ Hypertension was defined as a self-report of using anti-hypertensive medications within the previous 2 weeks, or a systolic BP ≥130 mmHg, or a diastolic BP ≥80 mmHg.

^b^ Hypertension was defined as a self-report of using anti-hypertensive medications within the previous 2 weeks, or a systolic BP ≥140 mmHg, or a diastolic BP ≥90 mmHg.

Abbreviation: BP, blood pressure.

**
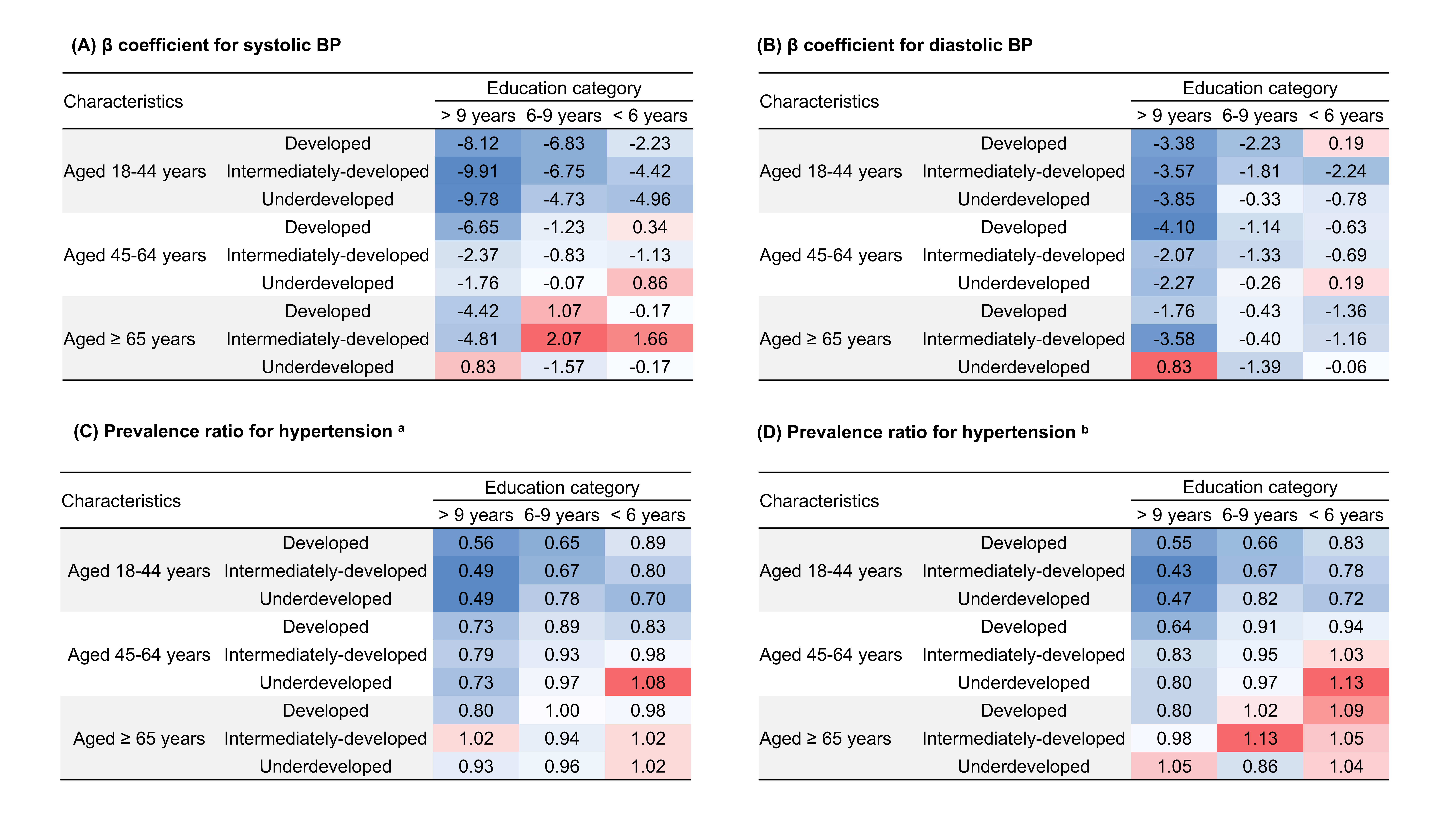
Figure S6.** Associations between sex and BP characteristics in subcategories of education duration and economic development status stratified by age groups after multivariable adjustment

Men were used as the reference group. Data are shown as β coefficients or prevalence ratios. The model was adjusted for smoking status, drinking status, physical activity, diet score, obesity, taking antihypertensive medications (in models for systolic BP and diastolic BP), and other SES indicators.

The changing blue and red colors depict the associations of women *vs.* men with higher levels of BP or hypertension prevalence. Darker blue indicates stronger inverse association and darker red indicates stronger positive association.

^a^ Hypertension was defined as a self-report of using anti-hypertensive medications within the previous 2 weeks, or a systolic BP ≥130 mmHg, or a diastolic BP ≥80 mmHg.

^b^ Hypertension was defined as a self-report of using anti-hypertensive medications within the previous 2 weeks, or a systolic BP ≥140 mmHg, or a diastolic BP ≥90 mmHg.

Abbreviation: BP, blood pressure; SES, socioeconomic status.

**
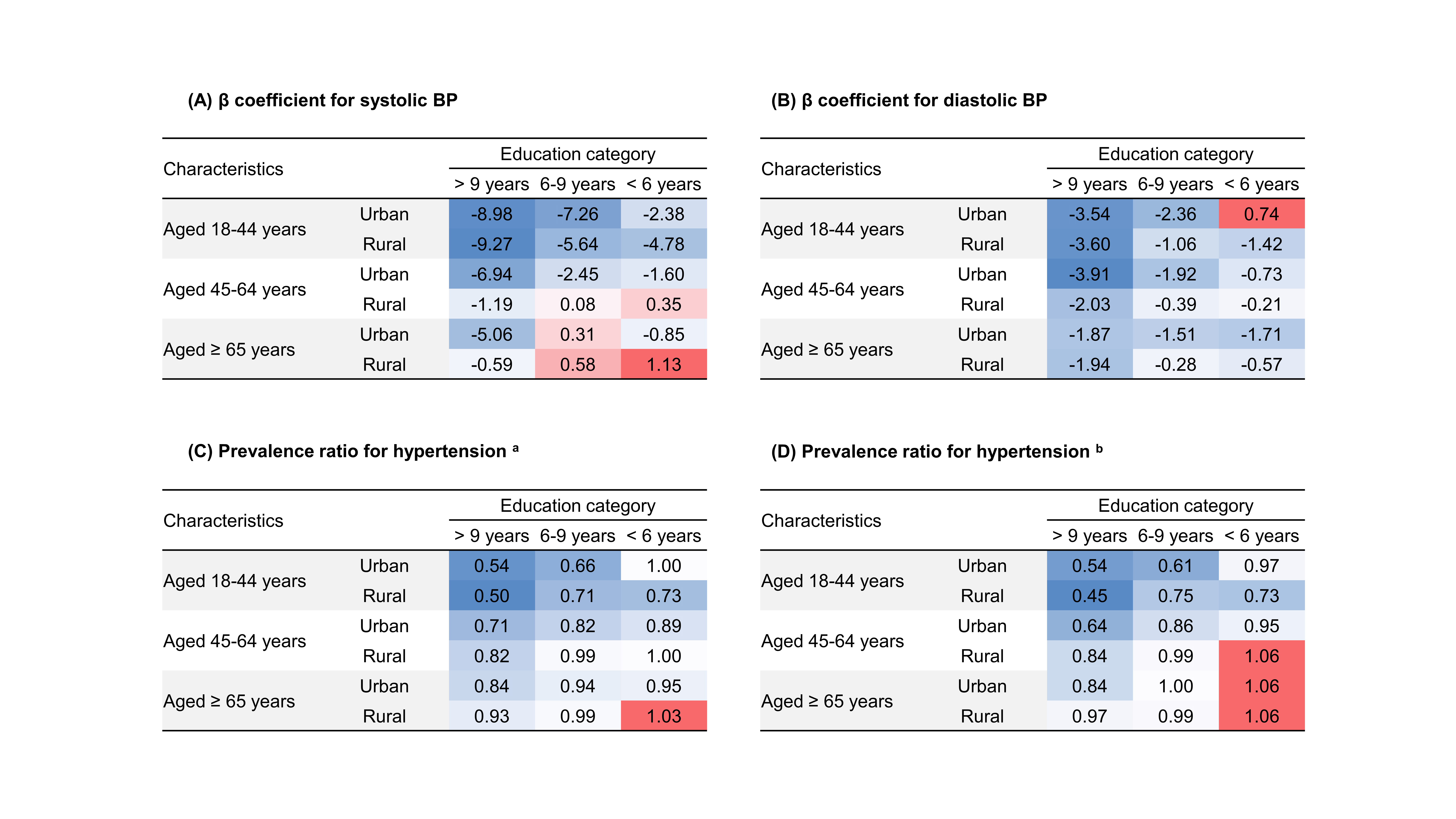
Figure S7**. Association between sex and BP characteristics in subcategories of education duration and area stratified by age groups after multivariable adjustment

Men were used as the reference group. Data are shown as β coefficients or prevalence ratios. The changing blue and red colors depict the associations of women *vs.* men with higher levels of BP or hypertension prevalence. Darker blue indicates stronger inverse association and darker red indicates stronger positive association.

The model was adjusted for smoking status, drinking status, physical activity, diet score, obesity, taking antihypertensive medications (in models for systolic BP and diastolic BP), and other SES indicators.

^a^ Hypertension was defined as a self-report of using anti-hypertensive medications within the previous 2 weeks, or a systolic BP ≥130 mmHg, or a diastolic BP ≥80 mmHg.

^b^ Hypertension was defined as a self-report of using anti-hypertensive medications within the previous 2 weeks, or a systolic BP ≥140 mmHg, or a diastolic BP ≥90 mmHg.

Abbreviation: BP, blood pressure; SES, socioeconomic status.

**
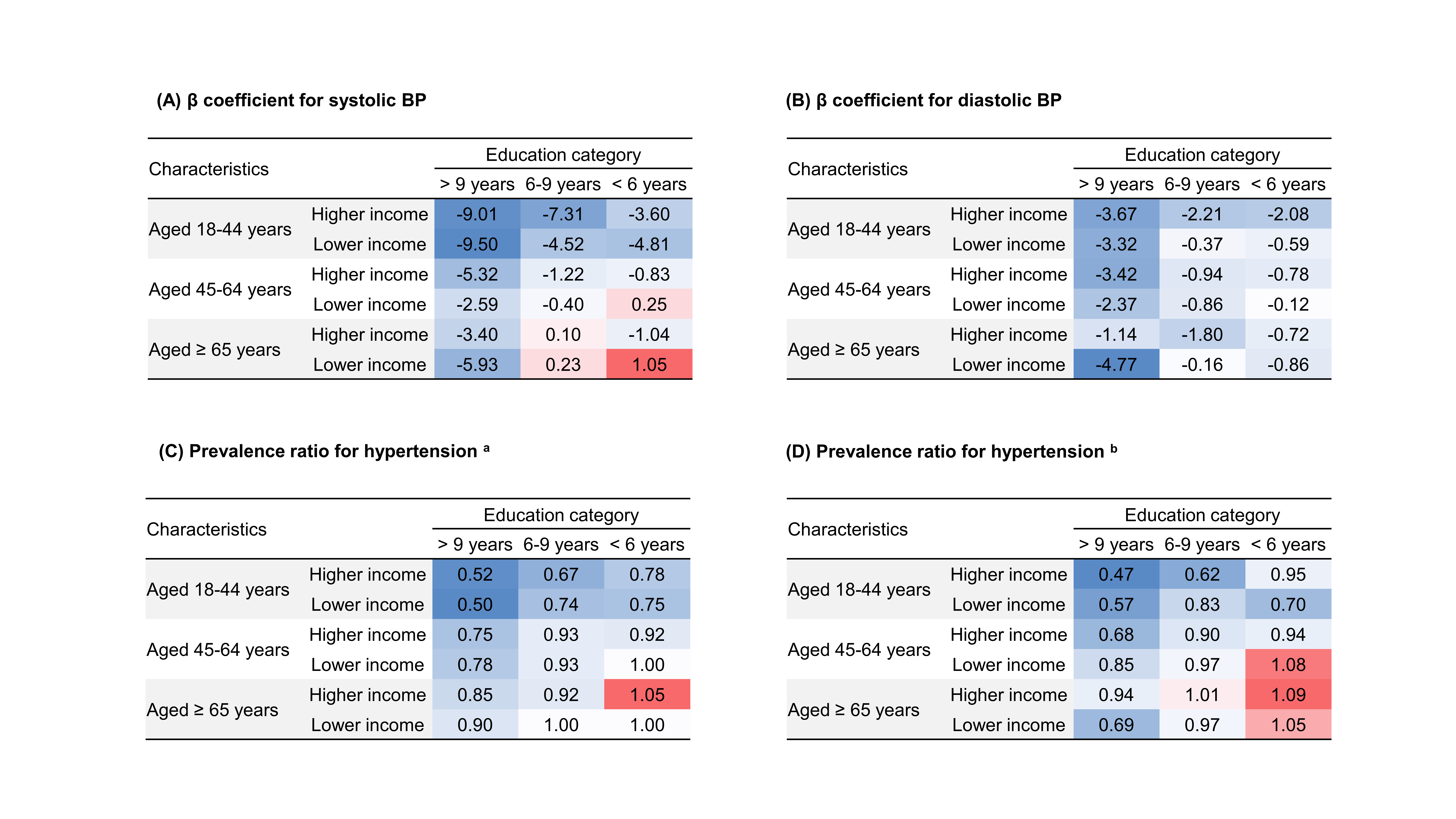
Figure S8**. Association between sex and BP characteristics in subcategories of education duration and annual household income stratified by age groups after multivariable adjustment

Men were used as the reference group. Data are shown as β coefficients or prevalence ratios. The changing blue and red colors depict the associations of women *vs.* men with higher levels of BP or hypertension prevalence. Darker blue indicates stronger inverse association and darker red indicates stronger positive association.

The model was adjusted for smoking status, drinking status, physical activity, diet score, obesity, taking antihypertensive medications (in models for systolic BP and diastolic BP), and other SES indicators.

^a^ Hypertension was defined as a self-report of using anti-hypertensive medications within the previous 2 weeks, or a systolic BP ≥130 mmHg, or a diastolic BP ≥80 mmHg.

^b^ Hypertension was defined as a self-report of using anti-hypertensive medications within the previous 2 weeks, or a systolic BP ≥140 mmHg, or a diastolic BP ≥90 mmHg.

Abbreviation: BP, blood pressure; SES, socioeconomic status.

**
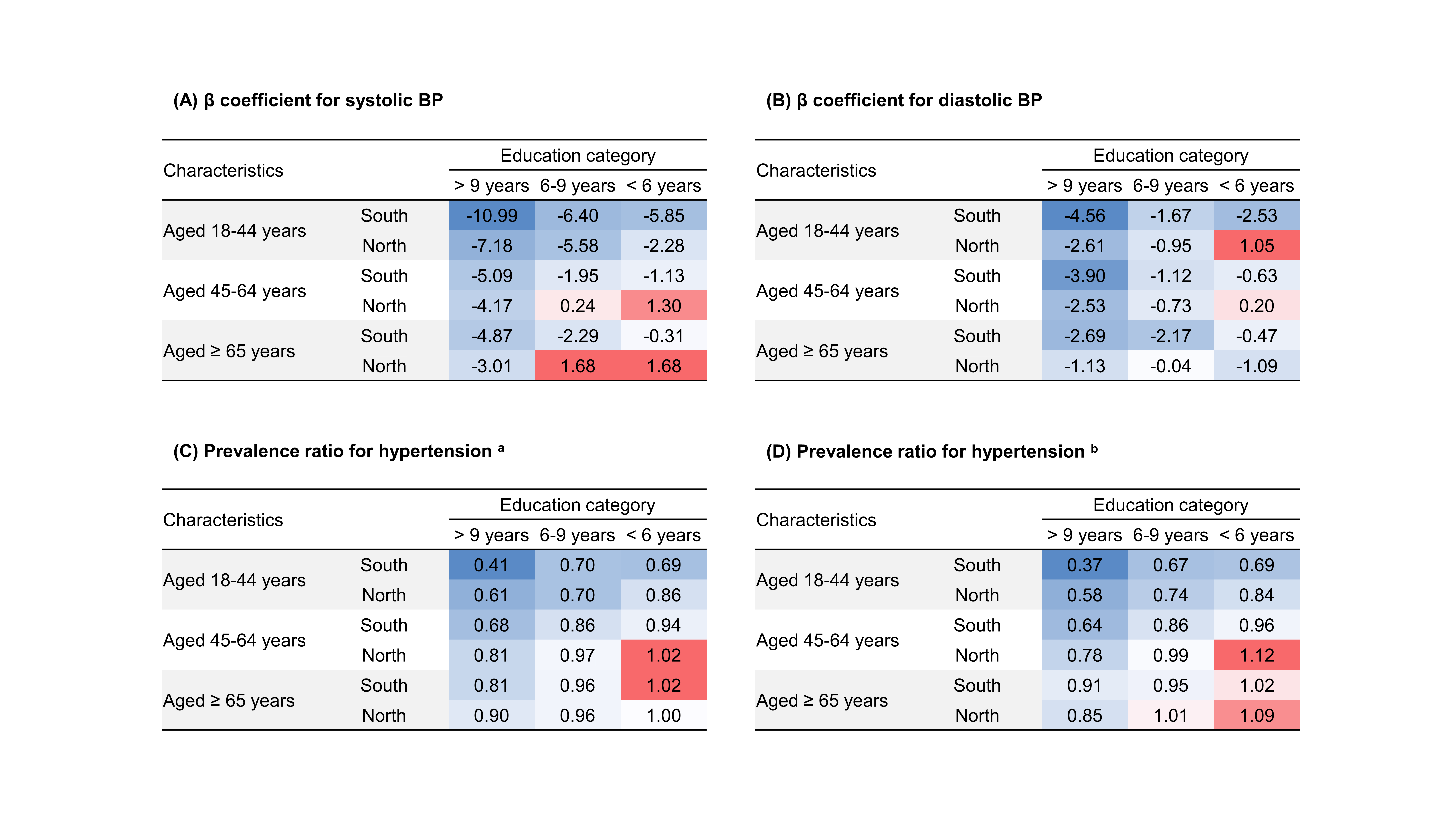
Figure S9**. Association between sex and BP characteristics in subcategories of education duration and region stratified by age groups after multivariable adjustment

Men were used as the reference group. Data are shown as β coefficients or prevalence ratios. The changing blue and red colors depict the associations of women *vs.* men with higher levels of BP or hypertension prevalence. Darker blue indicates stronger inverse association and darker red indicates stronger positive association.

The model was adjusted for smoking status, drinking status, physical activity, diet score, obesity, taking antihypertensive medications (in models for systolic BP and diastolic BP), and other SES indicators.

^a^ Hypertension was defined as a self-report of using anti-hypertensive medications within the previous 2 weeks, or a systolic BP ≥130 mmHg, or a diastolic BP ≥80 mmHg.

^b^ Hypertension was defined as a self-report of using anti-hypertensive medications within the previous 2 weeks, or a systolic BP ≥140 mmHg, or a diastolic BP ≥90 mmHg.

Abbreviation: BP, blood pressure; SES, socioeconomic status.

**
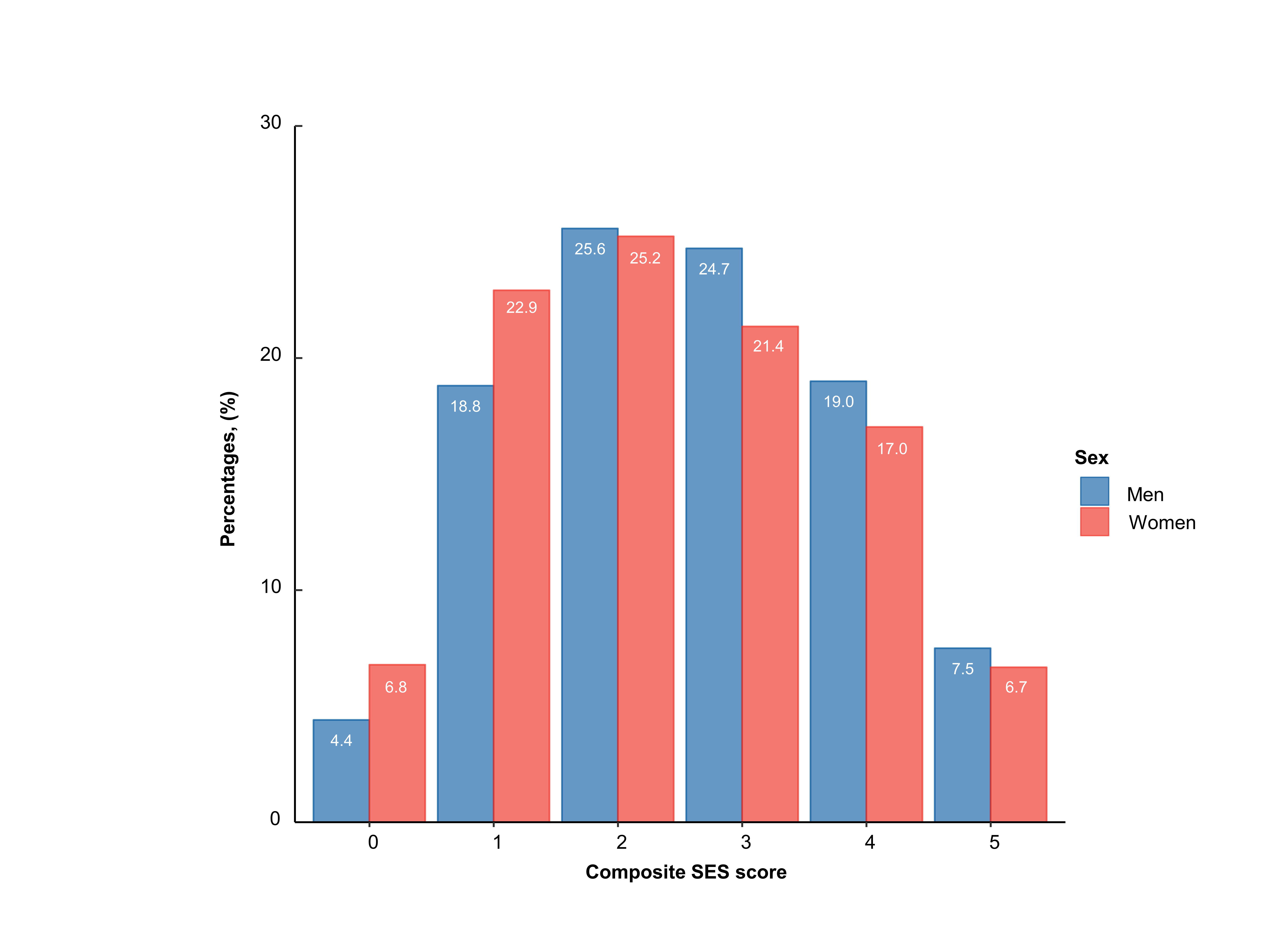
Figure S10**. Percentages of composite SES scores in Chinese men and women

Abbreviation: SES, socioeconomic status.

**
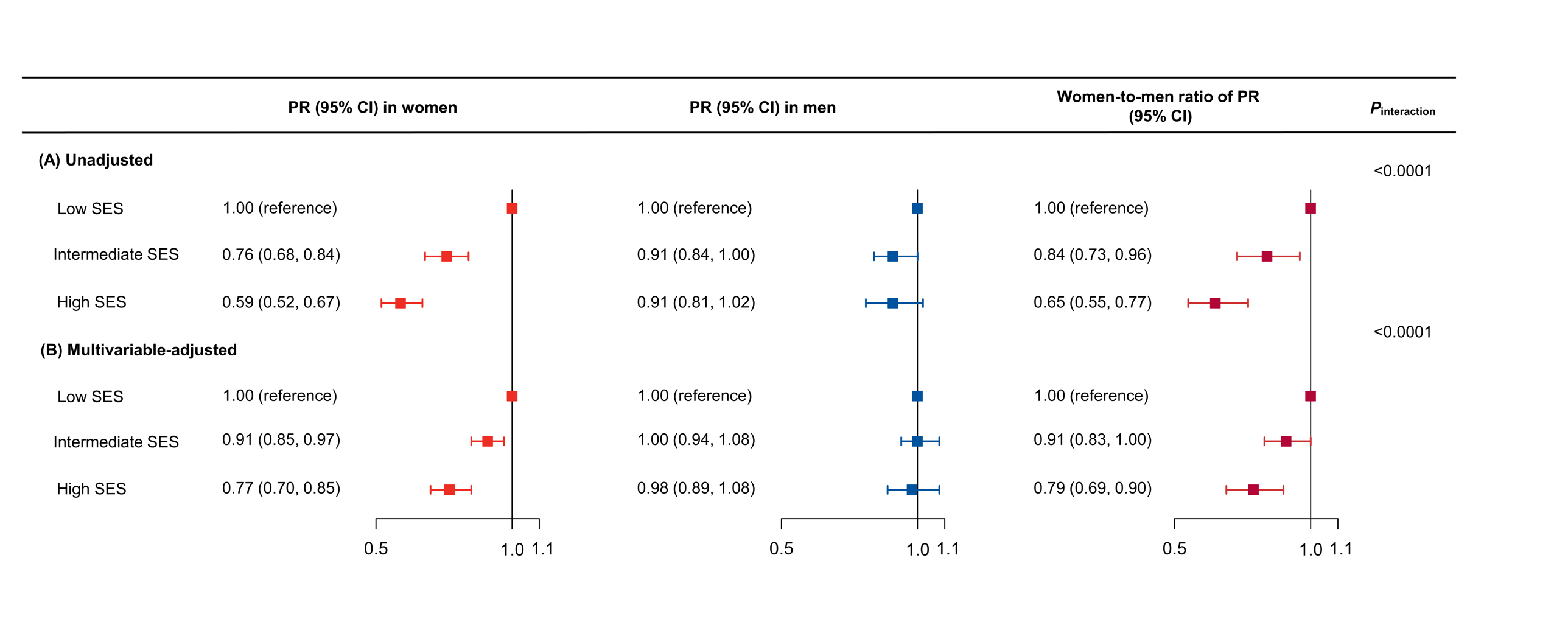
Figure S11.** Associations between the overall SES and prevalent hypertension defined by BP ≥130/80 mmHg in Chinese men and women in unadjusted (A) and multivariable-adjusted (B) models ^a^

The low SES group was the reference group. Data are shown as prevalence ratios (95% CIs). The multivariable model included age, smoking status, drinking status, physical activity, diet score, and obesity.

^a^ Hypertension was defined as a self-report of using anti-hypertensive medications within the previous 2 weeks, or a systolic BP ≥130 mmHg, or a diastolic BP ≥80 mmHg.

Abbreviation: SES, socioeconomic status; CI, confidence interval; BP, blood pressure; PR, prevalence ratio.

**Table S1.** Baseline characteristics of participants stratified by sex and education

| Characteristics | Men | | | *P* value | Women | | | *P* value |
| --- | --- | --- | --- | --- | --- | --- | --- | --- |
|  | Duration of education  <6 years | Duration of education  6-9 years | Duration of education  >9 years |  | Duration of education  <6 years | Duration of education  6-9 years | Duration of education  >9 years |  |
| Number of participants | 7568 | 25072 | 12503 |  | 16540 | 25271 | 11704 |  |
| Age (years) | 57.5 (56.2, 58.8) | 42.3 (41.3, 43.3) | 37.6 (36.7, 38.5) | <0.001 | 56.7 (55.5, 57.9) | 40.2 (39.2, 41.2) | 35.6 (34.2, 37.0) | <0.001 |
| Age category (%) |  |  |  | <0.001 |  |  |  | <0.001 |
| Aged 18-44 years | 23.5 (20.2, 26.8) | 59.9 (57.2, 62.7) | 69.2 (66.5, 71.9) |  | 23.8 (20.6, 27.0) | 66.6 (63.7, 69.4) | 73.9 (69.7, 78.1) |  |
| Aged 45-64 years | 42.7 (40.6, 44.7) | 32.1 (30.0, 34.3) | 27.0 (24.7, 29.3) |  | 46.0 (44.1, 47.9) | 28.1 (25.8, 30.4) | 23.6 (19.7, 27.5) |  |
| Aged ≥65 years | 33.8 (30.8, 36.8) | 7.9 (7.1, 8.8) | 3.8 (3.2, 4.5) |  | 30.2 (27.6, 32.8) | 5.3 (4.4, 6.2) | 2.5 (1.9, 3.2) |  |
| Current smoking (%) ^a^ | 54.7 (51.7, 57.7) | 55.7 (53.4, 58.0) | 47.9 (45.7, 50.1) | <0.001 | 3.7 (2.7, 4.8) | 2.4 (1.8, 3.0) | 1.2 (0.9, 1.6) | <0.001 |
| Excess drinking (%) ^b^ | 14.9 (13.0, 16.8) | 15.1 (13.6, 16.7) | 12.9 (11.4, 14.3) | 0.005 | 1.2 (0.8, 1.6) | 0.5 (0.3, 0.7) | 0.7 (0.5, 1.0) | 0.002 |
| Leisure-time physical activity (METs-h/week) ^c^ | 2.1 (1.6, 2.6) | 3.3 (2.8, 3.9) | 8.8 (7.8, 9.8) | <0.001 | 2.0 (1.5, 2.5) | 2.8 (2.2, 3.5) | 6.1 (5.2, 7.0) | <0.001 |
| Diet score >3 (%) | 0.5 (0.3, 0.8) | 1.1 (0.8, 1.3) | 2.8 (1.7, 3.9) | <0.001 | 0.6 (0.3, 0.8) | 1.3 (0.9, 1.8) | 3.9 (2.6, 5.1) | <0.001 |
| BMI (kg/m^2^) ^a^ | 23.0 (22.8, 23.1) | 23.7 (23.5, 23.9) | 24.2 (24.0, 24.4) | <0.001 | 24.0 (23.8, 24.2) | 23.8 (23.6, 24.0) | 23.0 (22.8, 23.3) | <0.001 |
| Waist circumference (cm) ^a^ | 80.0 (79.4, 80.7) | 81.9 (81.2, 82.6) | 83.5 (82.8, 84.3) | <0.001 | 80.0 (79.3, 80.7) | 78.4 (77.8, 79.0) | 76.0 (75.2, 76.8) | <0.001 |
| Obesity (%) ^a^ | 6.8 (5.7, 7.8) | 11.5 (10.4, 12.6) | 14.7 (13.2, 16.2) | <0.001 | 13.6 (12.3, 15.0) | 12.7 (11.5, 14.0) | 9.1 (7.8, 10.3) | <0.001 |
| Systolic BP (mmHg) ^a^ | 140.6 (139.0, 142.2) | 133.2 (132.1, 134.3) | 130.6 (129.5, 131.7) | <0.001 | 141.4 (139.5, 143.4) | 128.1 (126.8, 129.5) | 120.9 (119.4, 122.4) | <0.001 |
| Diastolic BP (mmHg) ^a^ | 82.5 (81.7, 83.2) | 81.4 (80.8, 82.1) | 81.6 (81.0, 82.3) | 0.001 | 83.0 (82.3, 83.7) | 79.8 (79.2, 80.4) | 77.1 (76.4, 77.8) | <0.001 |
| Prevalence of hypertension (%) ^a,d^ | 72.1 (69.2, 75.0) | 63.1 (60.5, 65.6) | 61.5 (58.8, 64.1) | <0.001 | 72.3 (69.3, 75.3) | 52.7 (50.1, 55.4) | 40.0 (36.8, 43.3) | <0.001 |
| Prevalence of hypertension (%) ^a,e^ | 48.7 (45.5, 52.0) | 34.7 (32.4, 37.1) | 30.5 (28.1, 32.9) | <0.001 | 51.7 (48.3, 55.1) | 28.2 (26.0, 30.4) | 17.6 (15.7, 19.6) | <0.001 |

Data are weighted means or percentages (95% confidence intervals).

^a^ There were 4 missing values for current smoking, 85 missing values for BMI, 75 missing values for waist circumference, 85 missing values for obesity, 67 missing values for systolic BP, 66 missing values for diastolic BP, and 62 missing values for status of hypertension.

^b^ Excess drinking was defined as currently having >21 drinks/week for men and >14 drinks/week for women.

^c^ Leisure-time physical activity was defined as the total of engaged vigorous or moderate physical activity at leisure time acquired from a questionnaire recording the frequency of the activities (days per week) and time spent doing the activities in a typical day.

^d^ Hypertension was defined as a self-report of using anti-hypertensive medications within the previous 2 weeks, or a systolic BP ≥130 mmHg, or a diastolic BP ≥80 mmHg.

^e^ Hypertension was defined as a self-report of using anti-hypertensive medications within the previous 2 weeks, or a systolic BP ≥140 mmHg, or a diastolic BP ≥90 mmHg.

Abbreviation: MET, metabolic equivalent; BMI, body mass index; BP, blood pressure.

**Table S2.** Baseline characteristics of participants stratified by sex and economic development status

| Characteristics | Men | | | *P* value | Women | | | *P* value |
| --- | --- | --- | --- | --- | --- | --- | --- | --- |
|  | Underdeveloped | Intermediately-developed | Developed |  | Underdeveloped | Intermediately-developed | Developed |  |
| Number of participants | 13373 | 16078 | 15692 |  | 15709 | 18664 | 19142 |  |
| Age (years) | 42.2 (40.6, 43.9) | 42.6 (41.5, 43.7) | 43.7 (42.7, 44.6) | 0.21 | 42.8 (41.0, 44.5) | 43.6 (42.3, 44.8) | 44.4 (43.2, 45.5) | 0.29 |
| Age category (%) |  |  |  | 0.14 |  |  |  | 0.11 |
| Aged 18-44 years | 60.4 (56.0, 64.7) | 58.8 (55.8, 61.8) | 55.1 (52.4, 57.8) |  | 59.8 (55.2, 64.4) | 57.2 (54.0, 60.3) | 53.5 (50.0, 57.1) |  |
| Aged 45-64 years | 30.4 (27.1, 33.6) | 31.1 (28.9, 33.3) | 34.4 (32.3, 36.5) |  | 29.5 (26.3, 32.7) | 31.3 (29.2, 33.5) | 34.8 (31.9, 37.8) |  |
| Aged ≥65 years | 9.3 (7.7, 10.8) | 10.2 (8.7, 11.7) | 10.5 (9.2, 11.8) |  | 10.8 (8.5, 13.0) | 11.5 (10.0, 13.1) | 11.6 (10.2, 13.1) |  |
| Current smoking (%) ^a^ | 54.2 (50.0, 58.4) | 53.0 (49.8, 56.1) | 52.6 (49.8, 55.4) | 0.78 | 2.1 (1.1, 3.0) | 2.9 (1.6, 4.1) | 2.5 (1.8, 3.1) | 0.50 |
| Excess drinking (%) ^b^ | 12.8 (10.3, 15.2) | 14.3 (12.5, 16.1) | 16.3 (14.0, 18.5) | 0.09 | 0.5 (0.2, 0.8) | 0.9 (0.5, 1.3) | 0.8 (0.5, 1.0) | 0.16 |
| Leisure-time physical activity (METs-h/week) ^c^ | 3.2 (2.5, 3.9) | 4.9 (3.8, 5.9) | 6.4 (5.1, 7.6) | <0.001 | 2.6 (1.6, 3.5) | 2.8 (2.1, 3.5) | 4.8 (3.6, 6.0) | 0.007 |
| Diet score >3 (%) | 0.7 (0.3, 1.2) | 1.1 (0.6, 1.6) | 2.7 (1.5, 3.9) | 0.002 | 0.8 (0.3, 1.4) | 1.2 (0.6, 1.7) | 3.2 (1.8, 4.6) | <0.001 |
| BMI (kg/m^2^) ^a^ | 23.2 (22.9, 23.5) | 23.7 (23.4, 23.9) | 24.5 (24.3, 24.7) | <0.001 | 23.3 (23.0, 23.6) | 23.6 (23.3, 23.9) | 24.2 (23.9, 24.4) | <0.001 |
| Waist circumference (cm) ^a^ | 80.1 (79.0, 81.3) | 81.7 (80.8, 82.7) | 84.6 (83.9, 85.2) | <0.001 | 77.1 (76.0, 78.2) | 78.0 (77.1, 78.9) | 79.6 (78.9, 80.4) | <0.001 |
| Obesity (%) ^a^ | 8.9 (7.3, 10.6) | 11.2 (9.6, 12.9) | 15.6 (14.1, 17) | <0.001 | 9.6 (8.0, 11.2) | 11.9 (10.2, 13.5) | 15.0 (13.2, 16.8) | <0.001 |
| Systolic BP (mmHg) ^a^ | 131.5 (129.8, 133.2) | 133.3 (131.7, 134.9) | 135.2 (133.8, 136.7) | 0.006 | 128.9 (126.3, 131.5) | 130.2 (128.0, 132.5) | 130.9 (129.0, 132.8) | 0.50 |
| Diastolic BP (mmHg) ^a^ | 79.8 (78.9, 80.6) | 81.9 (80.9, 82.8) | 83.2 (82.5, 83.9) | <0.001 | 78.9 (77.9, 79.9) | 80.2 (79.3, 81.2) | 80.9 (80.2, 81.6) | 0.006 |
| Prevalence of hypertension (%) ^a,d^ | 57.6 (53.5, 61.8) | 64.0 (60.6, 67.5) | 69.6 (66.8, 72.5) | <0.001 | 51.5 (46.9, 56.1) | 55.5 (51.4, 59.5) | 58.1 (55.2, 61.1) | 0.07 |
| Prevalence of hypertension (%) ^a,e^ | 30.1 (26.8, 33.5) | 35.0 (31.8, 38.2) | 40.7 (37.9, 43.4) | <0.001 | 29.0 (25.2, 32.8) | 32.3 (28.7, 35.8) | 34.8 (32.1, 37.6) | 0.06 |

Data are weighted means or percentages (95% confidence intervals).

^a^ There were 4 missing values for current smoking, 85 missing values for BMI, 75 missing values for waist circumference, 85 missing values for obesity, 67 missing values for systolic BP, 66 missing values for diastolic BP, and 62 missing values for status of hypertension.

^b^ Excess drinking was defined as currently having >21 drinks/week for men and >14 drinks/week for women.

^c^ Leisure-time physical activity was defined as the total of engaged vigorous or moderate physical activity at leisure time acquired from a questionnaire recording the frequency of the activities (days per week) and time spent doing the activities in a typical day.

^d^ Hypertension was defined as a self-report of using anti-hypertensive medications within the previous 2 weeks, or a systolic BP ≥130 mmHg, or a diastolic BP ≥80 mmHg.

^e^ Hypertension was defined as a self-report of using anti-hypertensive medications within the previous 2 weeks, or a systolic BP ≥140 mmHg, or a diastolic BP ≥90 mmHg.

Abbreviation: MET, metabolic equivalent; BMI, body mass index; BP, blood pressure.

**Table S3.** Baseline characteristics of participants stratified by sex and area

| Characteristics | Men | | *P* value | Women | | *P* value |
| --- | --- | --- | --- | --- | --- | --- |
|  | Urban | Rural |  | Urban | Rural |  |
| Number of participants | 17281 | 27862 |  | 21647 | 31868 |  |
| Age (years) | 44.0 (42.8, 45.2) | 42.3 (41.4, 43.2) | 0.03 | 44.7 (43.4, 46.0) | 43.0 (42.0, 44.0) | 0.049 |
| Age category (%) |  |  | 0.007 |  |  | 0.02 |
| Aged 18-44 years | 54.2 (50.9, 57.5) | 59.9 (57.4, 62.3) |  | 52.9 (49.0, 56.8) | 58.7 (56.0, 61.4) |  |
| Aged 45-64 years | 35.5 (33.1, 37.9) | 30.3 (28.5, 32.1) |  | 35.2 (32.2, 38.3) | 30.3 (28.4, 32.2) |  |
| Aged ≥65 years | 10.3 (8.8, 11.8) | 9.8 (8.8, 10.9) |  | 11.9 (10.1, 13.7) | 11.0 (9.8, 12.3) |  |
| Current smoking (%) ^a^ | 52.5 (49.5, 55.5) | 53.6 (51.1, 56.2) | 0.57 | 2.7 (2.0, 3.4) | 2.4 (1.6, 3.1) | 0.49 |
| Excess drinking (%) ^b^ | 13.8 (12.0, 15.6) | 14.7 (13.0, 16.4) | 0.47 | 0.7 (0.4, 0.9) | 0.8 (0.5, 1.0) | 0.66 |
| Leisure-time physical activity (METs-h/week) ^c^ | 7.1 (6.0, 8.2) | 3.8 (3.1, 4.4) | <0.001 | 5.6 (4.5, 6.6) | 2.4 (1.8, 2.9) | <0.001 |
| Diet score >3 (%) | 3.3 (2.0, 4.6) | 0.7 (0.4, 0.9) | <0.001 | 3.9 (2.5, 5.3) | 0.7 (0.4, 1.0) | <0.001 |
| BMI (kg/m^2^) ^a^ | 24.3 (24.1, 24.6) | 23.5 (23.3, 23.7) | <0.001 | 24.0 (23.7, 24.2) | 23.6 (23.3, 23.8) | 0.02 |
| Waist circumference (cm) ^a^ | 84.3 (83.3, 85.3) | 81.1 (80.4, 81.9) | <0.001 | 79.1 (78.2, 80.0) | 77.9 (77.2, 78.6) | 0.04 |
| Obesity (%) ^a^ | 14.7 (13.0, 16.5) | 10.6 (9.4, 11.8) | <0.001 | 13.7 (12.1, 15.4) | 11.4 (10.1, 12.7) | 0.03 |
| Systolic BP (mmHg) ^a^ | 133.7 (132.3, 135.1) | 133.2 (131.9, 134.5) | 0.62 | 128.8 (127.0, 130.6) | 130.6 (128.8, 132.3) | 0.17 |
| Diastolic BP (mmHg) ^a^ | 82.4 (81.7, 83.1) | 81.2 (80.5, 82.0) | 0.03 | 79.7 (79.0, 80.5) | 80.1 (79.4, 80.9) | 0.46 |
| Prevalence of hypertension (%) ^a,d^ | 65.9 (62.8, 69.0) | 62.8 (59.8, 65.8) | 0.16 | 53.8 (50.7, 57.0) | 55.5 (52.4, 58.6) | 0.47 |
| Prevalence of hypertension (%) ^a,e^ | 37.7 (34.7, 40.7) | 34.1 (31.5, 36.7) | 0.08 | 31.7 (29.0, 34.5) | 32.1 (29.4, 34.8) | 0.84 |

Data are weighted means or percentages (95% confidence intervals).

^a^ There were 4 missing values for current smoking, 85 missing values for BMI, 75 missing values for waist circumference, 85 missing values for obesity, 67 missing values for systolic BP, 66 missing values for diastolic BP, and 62 missing values for status of hypertension.

^b^ Excess drinking was defined as currently having >21 drinks/week for men and >14 drinks/week for women.

^c^ Leisure-time physical activity was defined as the total of engaged vigorous or moderate physical activity at leisure time acquired from a questionnaire recording the frequency of the activities (days per week) and time spent doing the activities in a typical day.

^d^ Hypertension was defined as a self-report of using anti-hypertensive medications within the previous 2 weeks, or a systolic BP ≥130 mmHg, or a diastolic BP ≥80 mmHg.

^e^ Hypertension was defined as a self-report of using anti-hypertensive medications within the previous 2 weeks, or a systolic BP ≥140 mmHg, or a diastolic BP ≥90 mmHg.

Abbreviation: MET, metabolic equivalent; BMI, body mass index; BP, blood pressure.

**Table S4.** Baseline characteristics of participants stratified by sex and annual household income

| Characteristics | Men | | *P* value | Women | | *P* value |
| --- | --- | --- | --- | --- | --- | --- |
|  | Higher income | Lower income |  | Higher income | Lower income |  |
| Number of participants | 23222 | 21921 |  | 26569 | 26946 |  |
| Age (years) | 40.1 (39.3, 41.0) | 46.0 (44.9, 47.1) | <0.001 | 40.4 (39.5, 41.3) | 46.8 (45.6, 47.9) | <0.001 |
| Age category (%) |  |  | <0.001 |  |  | <0.001 |
| Aged 18-44 years | 65.5 (63.1, 67.8) | 49.5 (46.6, 52.5) |  | 64.9 (62.1, 67.7) | 48.5 (45.6, 51.5) |  |
| Aged 45-64 years | 28.0 (26.3, 29.8) | 36.5 (34.3, 38.6) |  | 28.3 (26.1, 30.4) | 35.5 (33.5, 37.6) |  |
| Aged ≥65 years | 6.5 (5.6, 7.4) | 14.0 (12.6, 15.4) |  | 6.8 (5.9, 7.8) | 15.9 (14.3, 17.6) |  |
| Current smoking (%) ^a^ | 52.7 (50.6, 54.8) | 53.9 (51.1, 56.7) | 0.42 | 2.1 (1.5, 2.6) | 2.9 (2.2, 3.6) | 0.003 |
| Excess drinking (%) ^b^ | 14.4 (13.0, 15.7) | 14.5 (12.9, 16.2) | 0.82 | 0.7 (0.6, 0.9) | 0.7 (0.5, 1.0) | 0.84 |
| Leisure-time physical activity (METs-h/week) ^c^ | 6.0 (5.2, 6.8) | 3.4 (2.9, 3.9) | <0.001 | 4.2 (3.5, 4.9) | 2.5 (1.9, 3.1) | <0.001 |
| Diet score >3 (%) | 2.2 (1.5, 2.9) | 0.7 (0.4, 0.9) | <0.001 | 2.6 (1.7, 3.5) | 0.8 (0.5, 1.1) | <0.001 |
| BMI (kg/m^2^) ^a^ | 24.1 (23.9, 24.3) | 23.4 (23.2, 23.6) | <0.001 | 23.5 (23.3, 23.7) | 23.8 (23.6, 24.0) | 0.001 |
| Waist circumference (cm) ^a^ | 83.0 (82.4, 83.7) | 81.1 (80.3, 81.9) | <0.001 | 77.7 (77.1, 78.3) | 78.8 (78.1, 79.5) | 0.001 |
| Obesity (%) ^a^ | 13.5 (12.3, 14.8) | 10.0 (8.8, 11.1) | <0.001 | 11.4 (10.3, 12.4) | 12.9 (11.7, 14.1) | 0.002 |
| Systolic BP (mmHg) ^a^ | 132.0 (130.9, 133.0) | 134.9 (133.7, 136.2) | <0.001 | 126.4 (125.1, 127.6) | 133.8 (132.1, 135.4) | <0.001 |
| Diastolic BP (mmHg) ^a^ | 81.7 (81.1, 82.3) | 81.5 (80.7, 82.2) | 0.56 | 79.1 (78.6, 79.6) | 81.0 (80.2, 81.7) | <0.001 |
| Prevalence of hypertension (%) ^a,d^ | 62.7 (60.2, 65.2) | 64.9 (62.1, 67.8) | 0.11 | 49.9 (47.6, 52.3) | 60.2 (57.2, 63.3) | <0.001 |
| Prevalence of hypertension (%) ^a,e^ | 33.3 (31.0, 35.5) | 37.6 (34.9, 40.2) | 0.001 | 26.2 (24.4, 28.0) | 37.9 (35.1, 40.8) | <0.001 |

Data are weighted means or percentages (95% confidence intervals).

^a^ There were 4 missing values for current smoking, 85 missing values for BMI, 75 missing values for waist circumference, 85 missing values for obesity, 67 missing values for systolic BP, 66 missing values for diastolic BP, and 62 missing values for status of hypertension.

^b^ Excess drinking was defined as currently having >21 drinks/week for men and >14 drinks/week for women.

^c^ Leisure-time physical activity was defined as the total of engaged vigorous or moderate physical activity at leisure time acquired from a questionnaire recording the frequency of the activities (days per week) and time spent doing the activities in a typical day.

^d^ Hypertension was defined as a self-report of using anti-hypertensive medications within the previous 2 weeks, or a systolic BP ≥130 mmHg, or a diastolic BP ≥80 mmHg.

^e^ Hypertension was defined as a self-report of using anti-hypertensive medications within the previous 2 weeks, or a systolic BP ≥140 mmHg, or a diastolic BP ≥90 mmHg.

Abbreviation: MET, metabolic equivalent; BMI, body mass index; BP, blood pressure.

**Table S5.** Baseline characteristics of participants stratified by sex and region

| Characteristics | Men | | *P* value | Women | | *P* value |
| --- | --- | --- | --- | --- | --- | --- |
|  | South | North |  | South | North |  |
| Number of participants | 21557 | 23586 |  | 24850 | 28665 |  |
| Age (years) | 42.5 (41.3, 43.6) | 43.2 (42.3, 44.1) | 0.31 | 43.0 (41.7, 44.3) | 44.1 (43.2, 45.1) | 0.17 |
| Age category (%) |  |  | 0.14 |  |  | 0.17 |
| Aged 18-44 years | 59.6 (56.3, 62.8) | 56.5 (54.2, 58.9) |  | 58.6 (54.9, 62.3) | 55.0 (52.5, 57.6) |  |
| Aged 45-64 years | 31.2 (28.7, 33.7) | 32.7 (30.9, 34.4) |  | 30.6 (27.9, 33.3) | 33.2 (31.2, 35.1) |  |
| Aged ≥65 years | 9.2 (8.1, 10.4) | 10.8 (9.6, 12.0) |  | 10.8 (9.4, 12.3) | 11.8 (10.3, 13.3) |  |
| Current smoking (%) ^a^ | 56.4 (53.8, 58.9) | 50.0 (47.2, 52.7) | 0.001 | 1.6 (1.1, 2.1) | 3.4 (2.4, 4.4) | <0.001 |
| Excess drinking (%) ^b^ | 14.3 (12.5, 16.0) | 14.6 (12.7, 16.5) | 0.79 | 0.9 (0.6, 1.2) | 0.6 (0.4, 0.7) | 0.04 |
| Leisure-time physical activity (METs-h/week) ^c^ | 4.8 (4.2, 5.5) | 4.8 (3.7, 5.9) | 0.91 | 3.7 (2.9, 4.5) | 3.0 (2.2, 3.9) | 0.28 |
| Diet score >3 (%) | 1.6 (1.0, 2.3) | 1.3 (0.6, 2.1) | 0.56 | 2.1 (1.2, 3.0) | 1.3 (0.6, 2.1) | 0.21 |
| BMI (kg/m^2^) ^a^ | 23.2 (23.0, 23.4) | 24.4 (24.2, 24.6) | <0.001 | 23.0 (22.8, 23.2) | 24.4 (24.2, 24.6) | <0.001 |
| Waist circumference (cm) ^a^ | 80.3 (79.5, 81.1) | 84.1 (83.4, 84.9) | <0.001 | 76.4 (75.7, 77.1) | 80.2 (79.5, 80.8) | <0.001 |
| Obesity (%) ^a^ | 8.4 (7.5, 9.4) | 15.6 (14.2, 17.0) | <0.001 | 8.4 (7.5, 9.2) | 16.0 (14.7, 17.4) | <0.001 |
| Systolic BP (mmHg) ^a^ | 130.8 (129.5, 132.1) | 136.1 (135.0, 137.2) | <0.001 | 126.4 (124.6, 128.2) | 133.7 (132.4, 135.0) | <0.001 |
| Diastolic BP (mmHg) ^a^ | 80.5 (79.7, 81.2) | 82.8 (82.1, 83.6) | <0.001 | 78.5 (77.7, 79.2) | 81.6 (81.1, 82.1) | <0.001 |
| Prevalence of hypertension (%) ^a,d^ | 58.1 (55.0, 61.3) | 69.8 (67.4, 72.1) | <0.001 | 48.2 (45.0, 51.5) | 62.0 (59.9, 64.1) | <0.001 |
| Prevalence of hypertension (%) ^a,e^ | 30.2 (27.6, 32.8) | 40.7 (38.3, 43.1) | <0.001 | 26.6 (23.8, 29.4) | 37.6 (35.5, 39.8) | <0.001 |

Data are weighted means or percentages (95% confidence intervals).

^a^ There were 4 missing values for current smoking, 85 missing values for BMI, 75 missing values for waist circumference, 85 missing values for obesity, 67 missing values for systolic BP, 66 missing values for diastolic BP, and 62 missing values for status of hypertension.

^b^ Excess drinking was defined as currently having >21 drinks/week for men and >14 drinks/week for women.

^c^ Leisure-time physical activity was defined as the total of engaged vigorous or moderate physical activity at leisure time acquired from a questionnaire recording the frequency of the activities (days per week) and time spent doing the activities in a typical day.

^d^ Hypertension was defined as a self-report of using anti-hypertensive medications within the previous 2 weeks, or a systolic BP ≥130 mmHg, or a diastolic BP ≥80 mmHg.

^e^ Hypertension was defined as a self-report of using anti-hypertensive medications within the previous 2 weeks, or a systolic BP ≥140 mmHg, or a diastolic BP ≥90 mmHg.

Abbreviation: MET, metabolic equivalent; BMI, body mass index; BP, blood pressure.

**Table S6**. The association between sex and BP characteristics in different categories of SES in unadjusted models

|  | Systolic BP | |  | | Diastolic BP | | |  | Hypertension ^a^ | |
| --- | --- | --- | --- | --- | --- | --- | --- | --- | --- | --- |
|  | β coefficient  (95% CI) | *P*_interaction_ | |  | | β coefficient  (95% CI) | *P*_interaction_ |  | Prevalence ratio (95% CI) | *P*_interaction_ |
| **Education** |  |  | |  | |  |  |  |  |  |
| Duration of education <6 years | 0.82 (-0.37, 2.02) | <0.001 | |  | | 0.51 (-0.02, 1.04) | <0.001 |  | 1.09 (1.02, 1.16) | <0.001 |
| Duration of education 6-9 years | -5.04 (-5.80, -4.28) |  |  |  | | -1.63 (-2.06, -1.20) |  |  | 0.78 (0.73, 0.83) |  |
| Duration of education >9 years | -9.70 (-10.66, -8.74) |  |  |  | | -4.54 (-5.10, -3.99) |  |  | 0.53 (0.49, 0.59) |  |
| **Economic Development** |  |  | |  | |  |  |  |  |  |
| Underdeveloped | -2.58 (-3.88, -1.28) | 0.03 | |  | | -0.87 (-1.57, -0.18) | 0.001 |  | 0.95 (0.88, 1.04) | 0.006 |
| Intermediately-developed | -3.11 (-4.20, -2.01) |  |  |  | | -1.61 (-2.16, -1.06) |  |  | 0.90 (0.83, 0.98) |  |
| Developed | -4.33 (-5.24, -3.43) |  |  |  | | -2.31 (-2.77, -1.84) |  |  | 0.82 (0.77, 0.88) |  |
| **Area** |  |  | |  | |  |  |  |  |  |
| Urban | -4.89 (-5.76, -4.03) | <0.001 | |  | | -2.66 (-3.04, -2.28) | <0.001 |  | 0.81 (0.76, 0.86) | 0.001 |
| Rural | -2.63 (-3.47, -1.80) |  |  |  | | -1.12 (-1.58, -0.66) |  |  | 0.93 (0.88, 0.99) |  |
| **Annual Household Income** |  |  | |  | |  |  |  |  |  |
| Higher income | -5.62 (-6.33, -4.90) | <0.001 | |  | | -2.62 (-3.02, -2.21) | <0.001 |  | 0.75 (0.71, 0.80) | <0.001 |
| Lower income | -1.17 (-1.89, -0.45) |  |  |  | | -0.54 (-0.93, -0.14) |  |  | 1.01 (0.96, 1.06) |  |
| **Region** |  |  | |  | |  |  |  |  |  |
| South | -4.38 (-5.24, -3.53) | <0.001 | |  | | -2.01 (-2.45, -1.58) | 0.02 |  | 0.86 (0.81, 0.92) | 0.27 |
| North | -2.32 (-3.15, -1.49) |  |  |  | | -1.20 (-1.73, -0.68) |  |  | 0.91 (0.85, 0.97) |  |

Men were used as the reference group. β coefficients (95% CIs) or prevalence ratios (95% CIs) for women *vs.* men are presented.

^a^ Hypertension was defined as a self-report of using anti-hypertensive medications within the previous 2 weeks, or a systolic BP ≥140 mmHg, or a diastolic BP ≥90 mmHg.

Abbreviation: BP, blood pressure; SES, socioeconomic status; SE, standard error; CI, confidence interval.

**Table S7**. Prevalence of hypertension and the association between sex and hypertension defined by BP ≥130/80 mmHg in different categories of SES in unadjusted and multivariable-adjusted models

|  | Prevalence of Hypertension (%) ^a^ | | Hypertension ^a,b^ | | Hypertension ^a,c^ | | |
| --- | --- | --- | --- | --- | --- | --- | --- |
|  | Men | Women | Prevalence ratio (95% CI) | *P*_interaction_ | Prevalence ratio (95% CI) | *P*_interaction_ | |
| **Education** |  |  |  |  |  |  |  |
| Duration of education <6 years | 72.1 (69.2, 75.0) | 72.3 (69.3, 75.3) | 1.00 (0.95, 1.06) | <0.001 | 0.96 (0.92, 1.01) | <0.001 | |
| Duration of education 6-9 years | 63.1 (60.5, 65.6) * | 52.7 (50.1, 55.4) * | 0.76 (0.73, 0.80) |  | 0.80 (0.77, 0.84) |  |  |
| Duration of education >9 years | 61.5 (58.8, 64.1) * | 40.0 (36.8, 43.3) * | 0.55 (0.51, 0.59) |  | 0.61 (0.56, 0.66) |  |  |
| **Economic Development** | |  |  |  |  |  |  |
| Underdeveloped | 57.6 (53.5, 61.8) * | 51.5 (46.9, 56.1) * | 0.85 (0.80, 0.91) | 0.002 | 0.82 (0.76, 0.89) | 0.008 | |
| Intermediately-developed | 64.0 (60.6, 67.5) * | 55.5 (51.4, 59.5) * | 0.80 (0.76, 0.85) |  | 0.77 (0.72, 0.82) |  |  |
| Developed | 69.6 (66.8, 72.5) * | 58.1 (55.2, 61.1) * | 0.75 (0.72, 0.78) |  | 0.74 (0.70, 0.79) |  |  |
| **Area** |  |  |  |  |  |  |  |
| Urban | 65.9 (62.8, 69.0) * | 53.8 (50.7, 57.0) * | 0.74 (0.70, 0.77) | <0.001 | 0.72 (0.68, 0.76) | <0.001 | |
| Rural | 62.8 (59.8, 65.8) * | 55.5 (52.4, 58.6) * | 0.83 (0.80, 0.87) |  | 0.80 (0.77, 0.84) |  |  |
| **Annual Household Income** | |  |  |  |  |  |  |
| Higher income | 62.7 (60.2, 65.2) * | 49.9 (47.6, 52.3) * | 0.72 (0.69, 0.74) | <0.001 | 0.72 (0.69, 0.75) | <0.001 | |
| Lower income | 64.9 (62.1, 67.8) * | 60.2 (57.2, 63.3) * | 0.89 (0.85, 0.92) |  | 0.83 (0.79, 0.88) |  |  |
| **Region** |  |  |  |  |  |  |  |
| South | 58.1 (55.0, 61.3) * | 48.2 (45.0, 51.5) * | 0.77 (0.73, 0.80) | 0.02 | 0.73 (0.70, 0.77) | 0.001 | |
| North | 69.8 (67.4, 72.1) * | 62.0 (59.9, 64.1) * | 0.83 (0.79, 0.86) |  | 0.81 (0.76, 0.86) |  |  |

Data are weighted means or percentages (95% confidence intervals). Prevalence ratios (95% CIs) for women *vs.* men are presented, for which men were used as the reference group.

**P* <0.05 comparing BP characteristics between men and women.

^a^ Hypertension was defined as a self-report of using anti-hypertensive medications within the previous 2 weeks, or a systolic BP ≥130 mmHg, or a diastolic BP ≥80 mmHg.

^b^The model was unadjusted.

^c^The model was adjusted for age, smoking status, drinking status, physical activity, diet score, obesity, and other SES indicators in the table.

Abbreviation: BP, blood pressure; SES, socioeconomic status; SE, standard error; CI, confidence interval.
